# TREM2 alleviates white matter injury after traumatic brain injury in mice might be mediated by regulation of DHCR24/LXR pathway in microglia

**DOI:** 10.1002/ctm2.1665

**Published:** 2024-04-22

**Authors:** Zhao Li, Shenghui Yu, Lin Li, Chao Zhou, Lin Wang, Shuang Tang, Nina Gu, Zhaosi Zhang, Zhijian Huang, Hong Chen, Wei Tang, Yingwen Wang, Xiaomin Yang, Xiaochuan Sun, Jin Yan

**Affiliations:** ^1^ Department of Neurosurgery The First Affiliated Hospital of Chongqing Medical University Chongqing China; ^2^ Emergency Department Chengdu First People's Hospital Chengdu China; ^3^ Department of Neurosurgery Chongqing University Cancer Hospital Chongqing China; ^4^ Department of Neurosurgery Nanchong Central Hospital The Second Clinical Medical College of North Sichuan Medical College Nanchong China; ^5^ Department of Neurosurgery Suining Central Hospital Suining China

**Keywords:** DHCR24, LXR, microglia, oligodendrocyte precursor cells, oligodendrocytes, sterol metabolism, traumatic brain injury, TREM2, white matter injury

## Abstract

**Background:**

White matter injury (WMI) is an important pathological process after traumatic brain injury (TBI). The correlation between white matter functions and the myeloid cells expressing triggering receptor‐2 (TREM2) has been convincingly demonstrated. Moreover, a recent study revealed that microglial sterol metabolism is crucial for early remyelination after demyelinating diseases. However, the potential roles of TREM2 expression and microglial sterol metabolism in WMI after TBI have not yet been explored.

**Methods:**

Controlled cortical injury was induced in both wild‐type (WT) and TREM2 depletion (TREM2 KO) mice to simulate clinical TBI. COG1410 was used to upregulate TREM2, while PLX5622 and GSK2033 were used to deplete microglia and inhibit the liver X receptor (LXR), respectively. Immunofluorescence, Luxol fast blue staining, magnetic resonance imaging, transmission electron microscopy, and oil red O staining were employed to assess WMI after TBI. Neurological behaviour tests and electrophysiological recordings were utilized to evaluate cognitive functions following TBI. Microglial cell sorting and transcriptomic sequencing were utilized to identify alterations in microglial sterol metabolism‐related genes, while western blot was conducted to validate the findings.

**Results:**

TREM2 expressed highest at 3 days post‐TBI and was predominantly localized to microglial cells within the white matter. Depletion of TREM2 worsened aberrant neurological behaviours, and this phenomenon was mediated by the exacerbation of WMI, reduced renewal of oligodendrocytes, and impaired phagocytosis ability of microglia after TBI. Subsequently, the upregulation of TREM2 alleviated WMI, promoted oligodendrocyte regeneration, and ultimately facilitated the recovery of neurological behaviours after TBI. Finally, the expression of DHCR24 increased in TREM2 KO mice after TBI. Interestingly, TREM2 inhibited DHCR24 and upregulated members of the LXR pathway. Moreover, LXR inhibition could partially reverse the effects of TREM2 upregulation on electrophysiological activities.

**Conclusions:**

We demonstrate that TREM2 has the potential to alleviate WMI following TBI, possibly through the DHCR24/LXR pathway in microglia.

## Graphical abstract

TREM2 depletion exacerbates WMI and cognitive deficits after TBI. TREM2 upregulation alleviates WMI and promotes cognition recovery after TBI. DHCR24 is upregulated after TBI in TREM2 KO mice and can be inhibited by TREM2 upregulation. TREM2 may alleviate WMI after TBI by regulating DHCR24/LXR pathway in microglia.

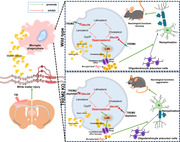

## 1 INTRODUCTION

Traumatic brain injury (TBI) refers to damage inflicted upon the brain by an external mechanical force, leading to neural injury and temporary or permanent functional impairment.^1^ In China, there are more TBI patients than in most other countries as a result of the large population and rapid economic development, posing a devastating burden on the economy and society.^2^ Thus, it is urgent to find new treatments that can effectively address TBI.

External mechanical forces directly strike the brain and induce primary brain injury, including direct brain tissue damage such as axonal damage, contusions, and hemorrhage.^3^ The initial mechanical damage leads to various biochemical cascades, including excessive glutamate release, free radical generation, neuroinflammatory responses, and other pathophysiological complications.^4^, ^5^, ^6^ Among these conditions, white matter injury (WMI) is a pivotal pathological process that occurs after TBI, which induces abnormalities in neural signal transmission and contributes to poor outcomes after TBI.^7^ Previous studies have demonstrated a strong correlation between WMI severity and neurological outcomes in patients with TBI. And WMI imaging has been performed to determine the prognosis of TBI.^7^, ^8^ WMI after TBI involves both initial traumatic axonal injury and secondary myelin pathology.^9^ In neurons, the volume of axons can be 10,000 times that of the parental neuronal cell body and this elongated structure can increase the vulnerability of axons to various mechanical injuries.^10^ Regrettably, there are currently no effective methods available for preventing or mitigating WMI after TBI.

Microglia are resident immune cells in the central nervous system (CNS), constituting approximately 12% of the total cellular population in the mouse brain. These cells are crucial for various biological functions, such as neural migration, neural survival, synaptic pruning, and oligodendrocyte myelination.^11^ Under pathological circumstances, microglia become activated, undergo proliferation, and migrate towards injured sites.^12^ After they reach the site of injury, microglia have two main biological functions: engulfing various types of tissue debris to clean up the environment and facilitating tissue repair by secreting cytokines, chemokines, and growth factors.^13^, ^14^ Specifically in demyelinating diseases, microglia can promote remyelination after injury via the clearance of debris and modulation of the extracellular microenvironment.^11^ Therefore, microglia‐mediated phagocytosis is critical for remyelination in various CNS injuries. Nevertheless, the molecular mechanisms governing the regulation of microglial phagocytosis remain inadequately understood.^15^ The clearance of myelin debris is a complicated process that includes the internalization of myelin debris, phagolysosome maturation, and cholesterol recycling.^16^, ^17^ In the adult brain, 70% of cholesterol is associated with myelin, which is a multilayer structure composed of proteins and lipid.^18^ Cholesterol plays a pivotal role in developmental myelination and remyelination after neurodegenerative diseases.^19^, ^20^ In the chronic stage of demyelinated diseases, oligodendrocytes and neuron‐derived cholesterol are essential for supporting remyelination.^21^, ^22^ However, microglia‐derived cholesterol is critical for early myelin repair in the acute phase of neurodegenerative diseases,^22^ indicating that sterol metabolism in microglia may also be important in other CNS pathologies. As a result, we investigated whether microglial sterol metabolism participates in myelin repair following TBI.

The triggering receptor‐2 (TREM2) is expressed mainly on microglia in the CNS.^23^, ^24^ Under normal conditions, TREM2 is essential for maintaining homeostasis across various systems. However, under pathological conditions, such as neurodegenerative and brain‐injured disorders, TREM2 confers that beneficial effects are widely accepted by reseachers.^25^ Individuals carrying TREM2 mutations manifest demyelination in white matter and experience an early onset of dementia, recognized as Nasu‐Hakola disease.^26^ In addition, TREM2 depletion has been reported to inhibit microglial expansion in response to demyelination.^27^ Furthermore, TREM2 deficiency diminishes the phagocytic activity involving apoptotic cells, cellular debris, lipoproteins, Aβ, and bacterial products.^25^ More importantly, TREM2 activation promotes myelin debris clearance and improves remyelination after multiple sclerosis.^24^ These findings reveal that TREM2 is strongly linked to demyelinating diseases. Recently, Nugent et al. reported that in a cuprizone‐induced demyelination model, microglia lacking TREM2 could effectively phagocytose myelin debris, yet they exhibited impaired clearance of cholesterol, leading to the accumulation of intracellular cholesterol; this inability to clear cholesterol might be mediated by the cholesterol transport system liver X receptor (LXR).^28^ However, little is known about whether these pathophysiological processes also occur in TBI.

Overall, microglial sterol metabolism is crucial to the pathophysiology of myelin degradation and subsequent remyelination. Additionally, in various demyelinating diseases, TREM2 has been shown to regulate microglial phagocytosis and cholesterol metabolism. Our previous study further demonstrated that TREM2 can be upregulated by an apoE mimic peptide (COG1410), which subsequently alleviates neural damage after TBI.^29^ Thus, we propose that TREM2 may mitigate WMI after TBI by regulating microglial sterol metabolism‐related biological processes.

## 2 METHODS AND MATERIALS

**FIGURE 1:**
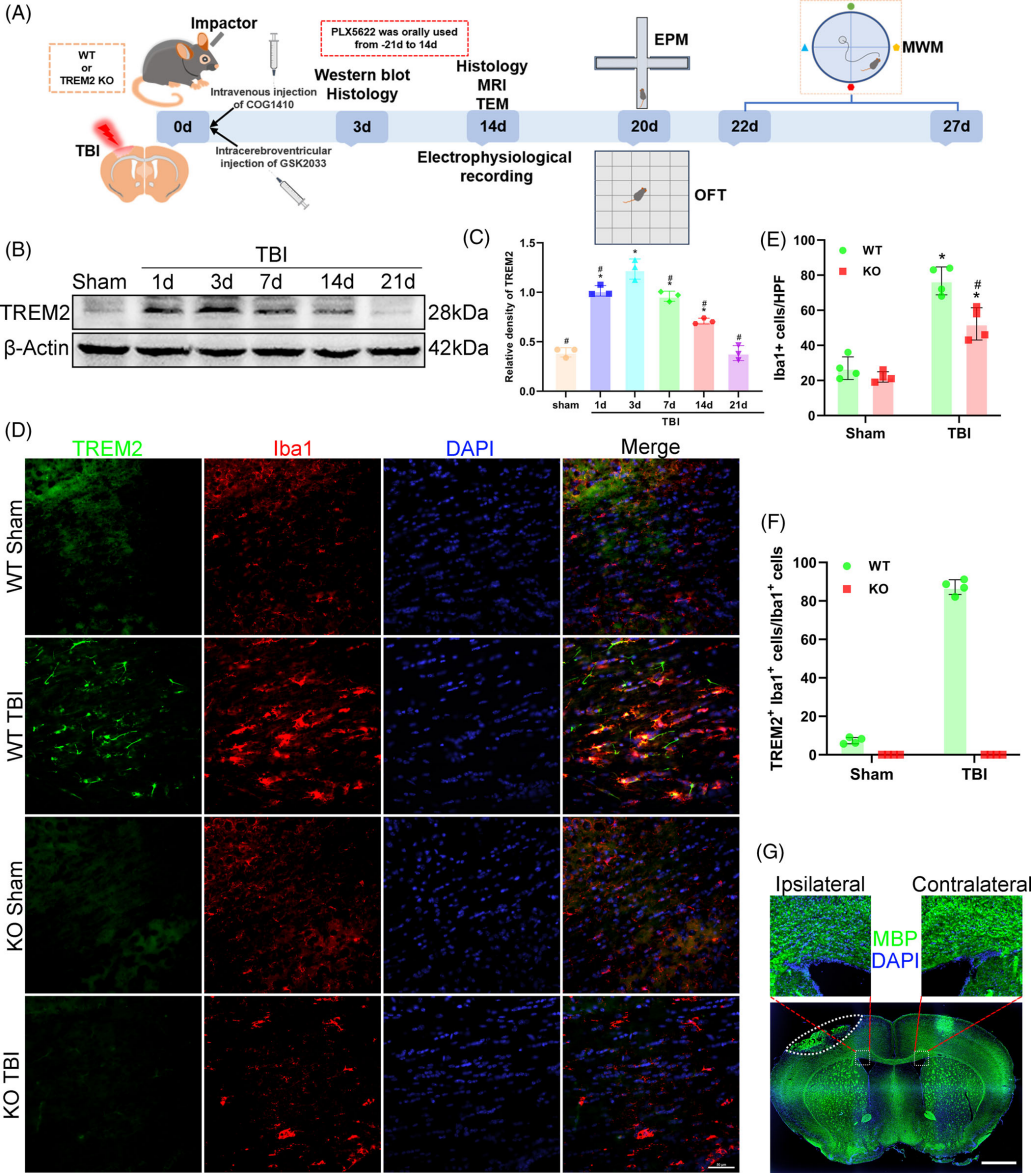
Time course of triggering receptor‐2 (TREM2) expression and cellular localization of TREM2 in the corpus callosum after traumatic brain injury (TBI). (A) Schematic diagram of experimental protocols and setups in this study. MRI, magnetic resonance imaging; TEM, transmission electron microscopy. (B) Representative western blot bands of TREM2 and β‐actin at different time points after TBI. (C) Quantitative analysis time course expression of TREM2 relative to β‐actin. ^*^ *p* < 0.05 versus Sham group, ^#^ *p* < 0.05 versus TBI 3d group; *n* = 3 per group. (D) Representative images of the colocalization of TREM2 (green) with microglia (Iba1, red) in corpus callosum area at 3 days after TBI. Nuclei were stained with DAPI (blue). Scale bar = 50 µm, *n* = 4. (E) Quantitative analysis of the number of microglia per high power field (HPF) in different groups. ^*^ *p *< 0.05 versus WT Sham group, ^#^ *p *< 0.05 versus WT TBI group; *n* = 4 per group. (F) Quantitative analysis of the percentage of TREM2^+^Iba1^+^ cells to Iba1^+^ cells, *n* = 4 per group. (G) Representative low magnification image showing myelin sheath loss (white square dashed box) and lesion site of TBI (white oval dashed box). The myelin sheath was stained by myelin basic protein (MBP) (green). Scale bar = 1000 µm.

All the experiments were performed in a blinded manner to avoid biases. Figure 1A presents a schematic diagram illustrating the protocols and setups employed in this study (Figure 1A).

### 2.1 Animals

The WT mice and the TREM2 KO mice were sourced from the Laboratory Animal Center of Chongqing Medical University and the Jackson Laboratory (#027197), respectively. The mice (8‐12 weeks old and approximately 25 g) were kept in a specific pathogen‐free environment with the conditions of suitable humidity and temperature, and a 12‐h light–dark cycle. The mice were allowed to have free access to food and water sources. As previously reported,^29^ the TREM2 KO mice were verified by using polymerase chain reaction (PCR), and the methods and results are shown in Figure S1. Microsoft Excel software was used to randomly assign the animals into groups. Throughout the whole experiment, all tools employed for animal procedures were sterilized using 75% alcohol, and the mice underwent deep anaesthesia with isoflurane (3% induction and 2% maintenance). Prior to brain tissue extraction, pentobarbital sodium (0.3%, 40 mg/kg) was used to induce deep anaesthesia in mice. In this study, a total of 402 mice (240 WT mice and 162 TREM2 KO mice) were used, and the detailed allocation of animals in the different experiments is shown in the figure legends.

### 2.2 TBI model induction

As previously reported, controlled cortical impact was induced to cause TBI in mice.^29^ Following the scalp incision, the mice were positioned and secured in a stereotaxic apparatus (RWD). Then, we drilled a hole with 4 mm diameter in the right cortex, where the centre was located at 2 mm (posterior) and 2.5 mm (lateral) relative to the bregma, and mechanical energy was applied using a pneumatic impactor (Jun‐Air Model 3−4). A cortical impact paradigm system (TBI‐0310, Fairfax) was employed to induce moderate TBI with the velocity was 5 m/s, the dwelling time was 100 ms, and the injured depth was 2 mm.^30^ A heating plate was used to maintain the body temperature of mice at approximately 37°C. Mice in the Sham group underwent a sham procedure without any mechanical insult.

### 2.3 Administration of drugs

As a previous study reported,^29^ a 0.2 mg/mL solution of COG1410 was delivered into mice through tail vein at 1 h after TBI. The total dose of COG1410 was 1 µg per gram of mouse's body weight and the COG1410 was dissolved in lactated Ringer's solution. In addition, 1200 ppm PLX5622 (#HY‐114153, MedChemExpress) was added to AIN‐76A standard chow to deplete microglia, and the chow was administrated starting 21 days before TBI induction and continuing until the end of the experiments.^31^, ^32^ In the LXR inhibition experiments, 5 µL of GSK2033 (#HY‐108688, MedChemExpress) was dissolved in 1% DMSO and administered via intracerebroventricular injection.^33^ For the vehicle‐treated groups, except for those in the LXR inhibition experiments, an equivalent volume of solution of lactated Ringer's was administrated via tail vein injection. For LXR inhibition experiments, an equivalent volume of lactated Ringer's solution was administered by tail vein injection; simultaneously, 5 µL of 1% DMSO was delivered by intracerebroventricular injection.

### 2.4 Western blot

For the analysis of TREM2 expression at various time points, total protein was extracted from the corpus callosum and external capsule by using a RIPA lysis buffer (Beyotime). Subsequently, the BCA assay was performed to measure total protein concentration. SDS‐polyacrylamide gel electrophoresis was performed to separate the proteins and the proteins were transferred onto PVDF membranes. The polyvinylidene fluoride (PVDF) membranes containing proteins were then incubated in fast blocking fluid for 20 min at room temperature to block excess antigen epitopes. After washing in Tris‐buffered saline/Tween‐20 (TBST), the proteins were incubated with primary antibodies at 4°C for 12 h. After washing in TBST, the suitable secondary antibody (Proteintech SA00001‐2, 1:10,000) was used to bind the primary antibodies for 1 h at 25°C. Raw protein bands were obtained by ECL chemiluminescence and the quantification of protein levels was conducted by ImageJ. The original western blot images are provided in Figures S3 and S4. The primary antibodies are shown in Supporting Information 1.

### 2.5 BrdU injections

On the third day post‐TBI, mice received intraperitoneal injections twice a day of the thymidine analogue BrdU (50 mg/kg, B9285, Sigma), with a minimum 8 h interval, over a span of four consecutive days.

### 2.6 Immunofluorescence staining

As previously outlined,^34^ following deep anaesthesia, mice were intracardially perfused with 50 mL cold saline and 50 mL cold 4% paraformaldehyde (PFA). The brains were then postfixed in PFA for 1 day, and dehydrated in 20% and 30% sucrose at 4°C. Subsequently, optimal cutting temperature was used to embed the brain, and 20‐µm‐thick coronal sections containing the corpus callosum were cut. After washing three times in PBS, the brain sections were embedded in a sodium citrate solution for 1 h at 95°C for antigen retrieval. After washing three times in PBS, the sections were permeabilized and blocked using fast blocking fluid at 25°C for 30 min. The sections were then incubated with the primary antibodies at 4°C for approximately 12 h. TUNEL staining was conducted to analyse cell death. After washing three times in PBS, the sections were incubated with suitable secondary antibodies for 1 h at 25°C. A Leica DM4 B fluorescence microscope was used to obtain all the immunofluorescent images, with consistent exposure times employed for quantitative analysis of each section. Colocalization analysis of different markers was performed using the Coloc 2 plugin in ImageJ software (ImageJ 1.4). Quantification of immunopositive cells and the immunostaining intensities of myelin basic protein (MBP) and SMI32 were conducted using ImageJ. The primary antibodies are shown in Supporting Information 1.

### 2.7 Neurobehavioral tests

#### 2.7.1 Open field test

As mentioned earlier with minor modifications,^29^ anxiety‐like behaviours were evaluated using the open field test (OFT) at 20 days after TBI. The spontaneous activities of mice in OFT were recorded for 5 min by ANY‐Maze software. The travelled time in centre and total distance were recorded and analysed.

#### 2.7.2 Elevated plus maze test

The elevated plus maze (EPM) was used to further test anxiety‐like behaviours after OFT was completed. The EPM apparatus included a cross‐shaped arena with two open arms and two closed arms, and the whole apparatus was placed 80 cm above the ground. The spontaneous activities of mice in EPM were recorded for 5 min by ANY‐Maze software. The travelled time in the open arms and the total travelled distance were recorded and analyzed.^35^

#### 2.7.3 Morris water maze test

As previously reported,^29^ Morris water maze (MWM) test was performed to assess cognitive functions. After the OFT and EPM tests, the mice were placed on a hidden platform in a pool approximately 1 cm under the water. This learning task was performed for two consecutive days. The navigation test was performed from 22 to 26 days after TBI. The latency that the mice spent finding the platform was recorded by ANY‐Maze from Stoelting. Finally, a probe trial was performed at 27 days after TBI, and the time spent in the quadrant where the platform was placed and the swimming speed in the pool were recorded and analysed.

### 2.8 Luxol fast blue staining

Myelin content and integrity were assessed using Luxol fast blue (LFB) staining, following previously described methods.^36^ Tissue slides were incubated with LFB from Sigma in acidified 95% alcohol at 60°C overnight. After being washed with 95% alcohol and ddH_2_O, the sections underwent immersion in a lithium carbonate solution, followed by exposure to a cresyl violet solution for differentiation and counterstaining. The images of LFB staining were acquired using a white light microscope, and the optical density was calculated by ImageJ.

### 2.9 Magnetic resonance imaging

Magnetic resonance imaging (MRI) was performed at 14 days post‐TBI to acquire diffusion tensor imaging (DTI) data for evaluating WMI. DTI data, including 5 A0 images and 30 noncolinear diffusion images, were obtained using a 7.0 T animal scanner with a multislice fast spin‐echo sequence from Bruker Biospin in Germany. ParaVision version 5.0 was used to analyse the DTI data. While blinded to group allocations, regions of interest encompassing the corpus callosum and external capsule in both the ipsilateral and contralateral hemispheres were manually delineated to determine fractional anisotropy (FA) values. The ParaVision version 5.0 was used again to calculate the values of directionally encoded colour and FA maps.^34^, ^37^

### 2.10 Transmission electron microscopy

Transmission electron microscopy (TEM) was conducted 14 days post‐TBI to measure myelin thickness in the corpus callosum. Mice underwent intracardial perfusion with cold saline and 2.5% glutaraldehyde, and subsequent careful dissection of the corpus callosum in the ipsilateral hemisphere into 1 mm^3^ blocks was performed. The blocks were subjected to overnight fixation in 2% glutaraldehyde at 4°C. After three times washing in PBS, the blocks were postfixed for 1 h in a mixed solution of 1% OsO_4_ and 1% K_3_Fe (CN)_6_. Subsequently, the tissue blocks underwent dehydration with graded alcohol and propylene oxide, followed by infiltration with mixture of propylene oxide and epoxy resin (1:1). The brain tissues were then embedded in resin within a mould. A Leica UCT ultramicrotome (Diatome) was used to cut the tissues into 60‐nm‐thick sections. These sections were mounted on copper grids, followed by staining with uranyl acetate and lead citrate. All the TEM images were obtained from randomly selected areas of the corpus callosum at a magnification of 20,000× using a JEM 1400Plus transmission electron microscope. The G‐ratio was calculated by dividing the inner diameter of the axon by the diameter of the entire axon.^37^

### 2.11 Oil red O staining

Oil red O (ORO) staining was conducted at 3 days after TBI to evaluate microglial phagocytosis.^38^ Brain sections underwent a 5 min dehydration in propylene glycol and were subsequently incubated with a 0.5% ORO solution from Sigma Aldrich (#O0625) for 15 min at 25°C. Subsequently, the brain sections were immersed in 85% propylene glycol for 5 min and subjected to three washes with distilled water. The images of ORO staining were acquired using a white light microscope, and the areas positive for ORO staining were analysed using ImageJ software.

### 2.12 Microglial magnetic sorting

As previously reported,^39^ after euthanasia, the mice underwent perfusion with cold PBS. Then, we extracted the brain tissues and used Hank's balanced salt solution to rinse them. Subsequently, the cortices and hippocampi were dissected from the brains of the mice, leaving behind white matter‐enriched tissues. Tissues from three mice were pooled to form a single sample. A Neural Tissue Dissociation Kit P was used to perform enzymatic cell dissociation (Miltenyi Biotec). Briefly, using papain to perform enzymatic digestion, the samples underwent mechanical dissociation, homogenization, and filtration using a 40 µm cell filter. After a wash in HBSS, myelin was removed by centrifuging. The isolated cells were incubated with CD11b MicroBeads for 15 min at 4°C. Consequently, the cells were suspended in 500 µL of PBS + 0.05% BSA. A magnetic column was used to purify the microglia. The verification of microglial purity is described in Figure S2.

### 2.13 Quantitative transcriptome analysis

RNA extraction from our sorted microglia was carried out using TRIzol reagent (Thermo Fisher Scientific, 15596018). Following a series of processing steps, we performed transcriptomics sequencing on an Illumina NovaSeq 6000 apparatus. In total, one million 2 × 150 bp paired‐end reads were obtained. These reads underwent additional filtering using Cutadapt 1.9 to obtain high‐quality clean reads.^40^ DESeq2 software and edgeR were used to conduct comparisons between two different groups and two samples, respectively, to obtain differential expression genes. Here, we extracted only the genes of interest for analysis (TREM2 and sterol‐associated genes). The complete transcriptome data are presented in Supporting Information 2.

### 2.14 Electrophysiological recording

#### 2.14.1 Motor‐evoked potential

As previously reported,^29^ Mice were positioned prone on a flat plate. Two 30‐G electrodes stimulating electrodes and a recording electrode were placed into the bilateral motor cortex and the left gastrocnemius muscle, respectively. An evoked potential instrument (Keypoint) was used to provide electrical stimulation and the stimulation was made up with a single pulse (7 mA, 1 ms, and 1 Hz). The electrical stimulation was performed five times for each mouse, with a 15 s interval between each repetition. The motor‐evoked potentials (MEPs) were recorded and the latency and amplitude were analysed.

#### 2.14.2 Local field potential

Five days prior to recording, a tungsten wire (A‐M systems, #795500) was positioned into the CA1 on the contralateral hemisphere of the mice and fixed using screws and dental cement. Following the electrode placement, mice were given 5 days to recovery before recording. While recording local field potential (LFP), all mice were granted the freedom moving in their home cages. Then, the LFP were recorded for a consecutive 5 min using a multichannel electrophysiological system (CerePlex Direct). The power spectral density of theta oscillations were calculated in the range of 4−12 Hz by using NeuroExplorer in the last 3 min of LFP data.^29^

### 2.15 Statistical analysis

**FIGURE 2:**
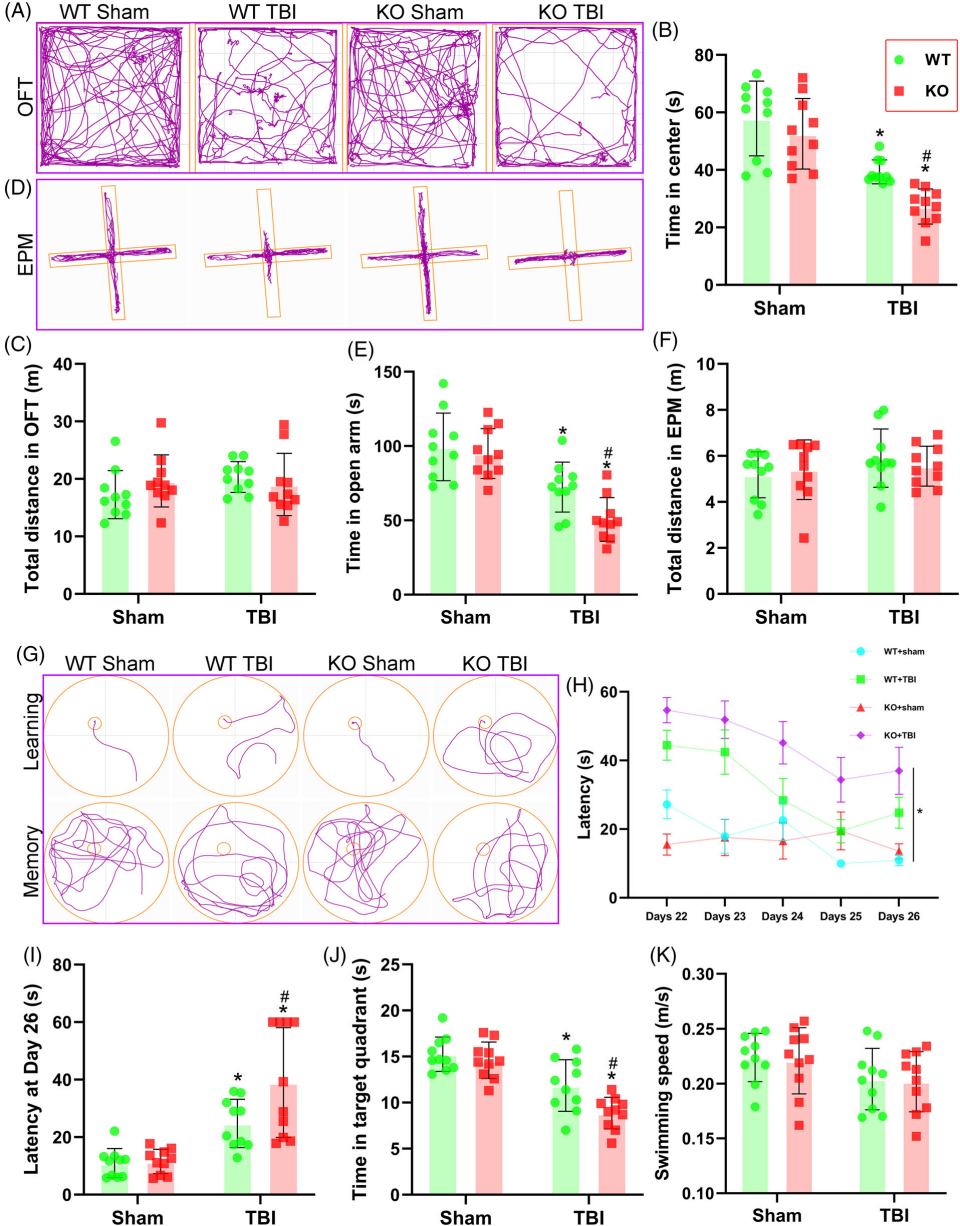
Triggering receptor‐2 (TREM2) depletion exacerbated long‐term cognitive dysfunction after traumatic brain injury (TBI). (A and D) Representative images of the trajectory of mice in open field test (OFT) (A) and elevated plus maze (EPM) (D) at 20 days after TBI. (B and C) Quantitative analysis of centre time (B) and total distance (C) travelled by mice in the OFT. (E and F) Quantitative analysis of open arms time (E) and total distance (F) travelled by mice in the EPM. (G) Representative images of the trajectory of mice in the Morris water maze (MWM). (H–K) Quantitative analysis of latency in the learning test (H and I), target quadrant time (J), and average swimming speed in the target quadrant test (K). ^*^ *p *< 0.05 versus WT Sham group, ^#^ *p *< 0.05 versus WT TBI group; *n* = 10 per group. ^*^ *p *< 0.05 in H represents analysis of variance (ANOVA) results.

In the present study, most of the results were presented as means ± standard deviations (SDs). In Figures 2H and 7H, the data were presented as means ± standard errors of the means (SEMs). At the beginning of statistical analyses, the normality of the data was evaluated by Shapiro–Wilk test. The unpaired Student's *t*‐test was used to compare the differences between two independent samples. Repeated‐measures analysis of variance (ANOVA) was conducted to analyse continuously measured data. When the ANOVA was significant, Tukey's post hoc multiple‐comparisons test was performed to display whether there are differences between two samples. Two‐way ANOVA with Tukey's post hoc multiple comparisons test was performed to compare the differences (WT Sham, WT TBI, TREM2 KO Sham, and TREM2 KO TBI). For comparisons involving other groups, one‐way ANOVA with Tukey's post hoc multiple comparisons test was used. GraphPad Prism (version 9.1.0) was used to conduct all the analyses. *p* < 0.05 was considered to achieve statistical significance.

## 3 RESULTS

### 3.1 Time course of TREM2 expression and cellular localization of TREM2 in the corpus callosum after TBI

To detect changes in TREM2 expression in the corpus callosum over time, we extracted corpus callosum tissue from the Sham mice at 3 days post‐sham operation, and at 1, 3, 7, 14, and 21 days after TBI. The western blot results demonstrated that the expression of TREM2 was significantly greater in the TBI mice at 1, 3, 7, and 14 days than in the Sham mice. Specifically, the TREM2 expression peaked at 3 days in the corpus callosum after TBI (*p *< 0.05, Figure 1B,C). Consequently, 3 days after TBI was considered as the main time point for our subsequent experiments. In addition, given that TREM2 is expressed mainly on microglia, we stained for TREM2 and Iba1 and found that the number of microglia was significantly increased in TBI groups than in the WT Sham mice (*p *< 0.05, Figure 1D,E). No significances in the number of microglia were detected between the two Sham groups (*p *> 0.05, Figure 1D,E). In addition, the number of microglia was significantly lower in the KO TBI mice when compared with the WT TBI mice (*p *< 0.05, Figure 1D,E), demonstrating that TREM2 is important for microglial activation. In addition, approximately 80% of the microglia was expressed TREM2, while in the Sham group, almost no microglia expressed TREM2 (Figure 1D,F). Moreover, almost all the TREM2^+^ cells were microglia (Figure 1D). In this study, all the histological observation areas were in the ipsilateral corpus callosum of the TBI sites, and MBP staining indicated a decreased fluorescence intensity on the ipsilateral side compared with the contralateral side (Figure 1G). However, the wide field images here cannot quantify the fluorescence of MBP due to uneven scanning.

### 3.2 TREM2 depletion exacerbated long‐term cognitive dysfunction after TBI

To investigate the impacts of TREM2 on neurological function post‐TBI, the OFT, EPM tests, and MWM test were performed at 3 weeks after TBI. In the OFT, we found that the TBI mice exhibited significant anxiety‐like behaviours, as indicated by shorter time spent in exploring the centre zone (*p *< 0.05, Figure 2A,B). Furthermore, compared with WT TBI mice, KO TBI mice spent less time exploring the central zone (*p *< 0.05, Figure 2A,B). Nevertheless, motor function, measured as total distance travelled, was not different among the four experimental groups (*p *> 0.05, Figure 2C), implying that the mice had no motor deficits at 3 weeks after TBI. Similarly, the EPM test results revealed the TBI mice spent less time exploring in the open arms (*p *< 0.05, Figure 2D,E), and TREM2 KO TBI mice exhibited a significant reduction in the exploring time in the open arms compared with the WT TBI mice (*p *< 0.05, Figure 2D,E). Once more, our data indicated that no motor deficits among these four groups at 3 weeks after TBI (*p *> 0.05, Figure 2F).

Next, during the learning stage in MWM test, we found that mice in the WT TBI group spent more time finding the hidden platform under the water compared to the WT Sham group (*p* < 0.05, Figure 2G–I). Furthermore, TREM2 KO TBI mice spent even more time finding the platform compared with the WT TBI mice (*p* < 0.05, Figure 2G–I). Additionally, in the memory test, the WT TBI group spent less time in the target quadrant after the removal of the hidden platform than that in the WT Sham group (*p* < 0.05, Figure 2J). Furthermore, compared with WT TBI group, TREM2 KO TBI mice showed further impairment in memory function, as evidenced by a shorter duration in the right quadrant (*p* < 0.05, Figure 2J). Importantly, all four groups of mice displayed normal motor skills, and no differences in swimming speed were observed during the memory stage (*p* > 0.05, Figure 2K). Interestingly, our findings demonstrated that TREM2 KO did not lead to significant changes in the cognitive functions of mice without TBI challenge (*p* > 0.05, Figure 2).

### 3.3 Genetic deletion of TREM2 aggravated white matter damage after TBI

**FIGURE 3:**
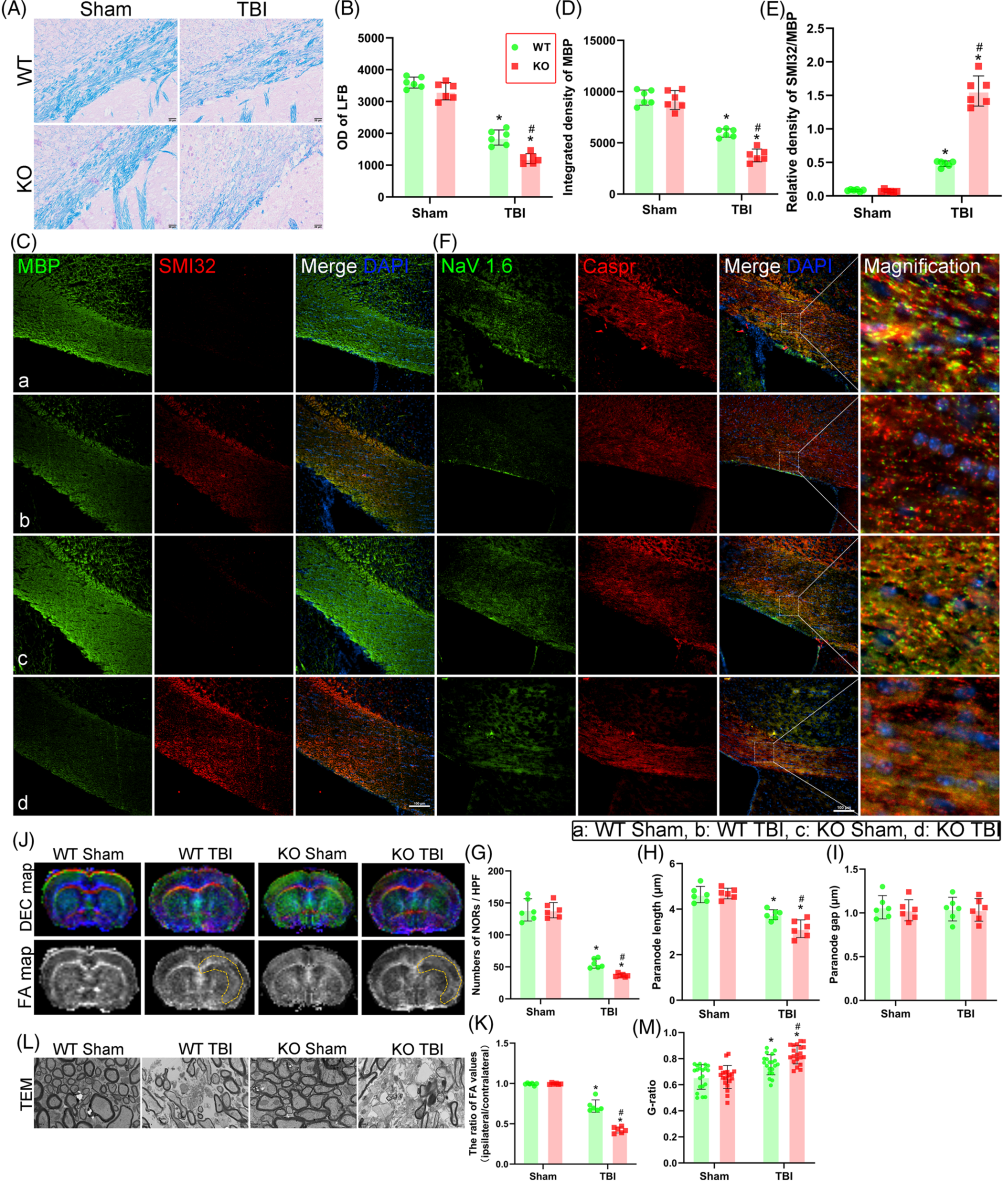
Genetic deletion of triggering receptor‐2 (TREM2) aggravated white matter damage after traumatic brain injury (TBI). (A) Representative images of the Luxol fast blue (LFB) in corpus callosum area at 3 days after TBI. Scale bar = 20 µm. (B) Quantitative analysis of the optical density in LFB staining. (C) Representative images of the colocalization of myelin basic protein (MBP) (green) with SMI32 (red) in corpus callosum area at 3 days after TBI. Nuclei were stained with DAPI (blue). Scale bar = 100 µm. (D and E) Quantitative analysis of the integrated density of MBP (D) and relative density of SMI32 to MBP (E). (F) Representative images of the colocalization of Nav1.6 (green) with Caspr (red) in corpus callosum area at 3 days after TBI. Nuclei were stained with DAPI (blue). Scale bar = 100 µm. (G–I) Quantitative analysis of number of the nodes of Ranvier (NOR) per high power field (HPF) (G), paranode length (H), and paranode gap in different groups (I). (J) Upper panel shows representative directionally encoded colour (DEC) maps centred on the site of contusion. Down panel shows representative fractional anisotropy (FA) values maps centred on the site of contusion. (K) Quantification of the ratio of FA values of the ipsilateral hemispheres to the contralateral hemispheres in the corpus callosum and external capsule (yellow dashed box). (L) Representative transmission electron microscopy (TEM) images of white matter tracts in corpus callosum region. (M) Quantitative analysis of the G‐ration in TEM experiments. Groups allocation is shown in the black solid line box. ^*^ *p *< 0.05 versus WT Sham group, ^#^ *p *< 0.05 versus WT TBI group; *n* = 6 per group.

Considering that TREM2 depletion exacerbated long‐term cognitive dysfunction after TBI, we wondered whether this effect was mediated by WMI after TBI. We evaluated WMI at 3 and 14 days after TBI. First, LFB staining was performed to assess myelin content and integrity at 3 days after TBI. We found that Sham‐treated mice had continuous and organized myelin structures, while TBI‐treated mice had discontinuous and disorganized myelin structures, indicating that the myelin was damaged by TBI (Figure 3A). Moreover, our statistical analysis revealed no differences in the optical density of LFB staining between the two Sham groups (*p *> 0.05, Figure 3B), and all the optical densities of the Sham groups were higher than those in the TBI groups (*p *< 0.05, Figure 3B). Besides, TREM2 KO further damaged the structure of the myelin sheath, as indicated by a more severely discontinuous and disorganized myelin structure, and the optical density of LFB staining was lower in the KO TBI mice than in the WT TBI mice (*p *< 0.05, Figure 3B).

MBP is crucial for maintaining the structure and function of the myelin sheath and exhibits specificity for neural tissue.^41^ Previous studies have showed that nonphosphorylated neurofilaments (SMI32‐positive) are increased after WMI.^42^ The ratio of SMI32 relative to MBP is usually used to reflect the severity of WMI in patients with various diseases.^43^ In this study, we found a significant decrease in the MBP density in the WT TBI mice compared to the WT Sham mice, and this decrease was further pronounced by TREM2 KO (*p* < 0.05, Figure 3C,D). Additionally, the WT TBI mice exhibited an elevated SMI32/MBP ratio compared to the WT Sham mice (*p* < 0.05, Figure 3C,E), indicating white matter injury. Remarkably, TREM2 deletion in TBI mice exacerbated WMI compared to WT controls, as evidenced by a further increase in the SMI32/MBP ratio (*p* < 0.05, Figure 3C,E). The examination of nodes of Ranvier (NORs) involved coimmunostaining for contactin‐associated protein (Caspr) and the Nav1.6 channel. Parameters such as the number of NORs, the paranode length, and the length of the paranode gap in the corpus callosum were quantified based on 15–20 NORs in each brain.^44^ The results showed that TBI elicited a significant decrease in the number of NORs (*p *< 0.05, Figure 3F,G) and paranode length (*p *< 0.05, Figure 3F,H) in WT mice at 3 days after TBI compared with those in WT Sham mice. In addition, the genetic deletion of TREM2 further reduced the number of NORs and shortened the paranode length in TBI groups, suggesting that NOR function was impaired (*p *< 0.05, Figure 3F–H). However, there were no significant differences in the length of the paranode gap among the four experimental groups (*p *> 0.05, Figure 3F,I).

Two weeks post‐TBI, DTI and TEM were employed to assess white matter integrity and measure myelin thickness, respectively. The results suggested that the WT TBI group had an exacerbated WMI, as indicated by increased FA values (*p *< 0.05, Figure 3J,K) and a decreased G‐ratio (*p *< 0.05, Figure 3L,M) when compared with the WT Sham controls. Most importantly, KO TBI mice exhibited further aggravated WMI when compared with the WT TBI mice (*p *< 0.05, Figure 3J–M). Throughout all the immunofluorescence staining tests for WMI, TREM2 deficiency did not affect myelin thickness when TBI was not induced.

### 3.4 TREM2 KO promoted oligodendrocyte apoptosis and suppressed oligodendrocyte and oligodendrocyte precursor cell proliferation after TBI

**FIGURE 4:**
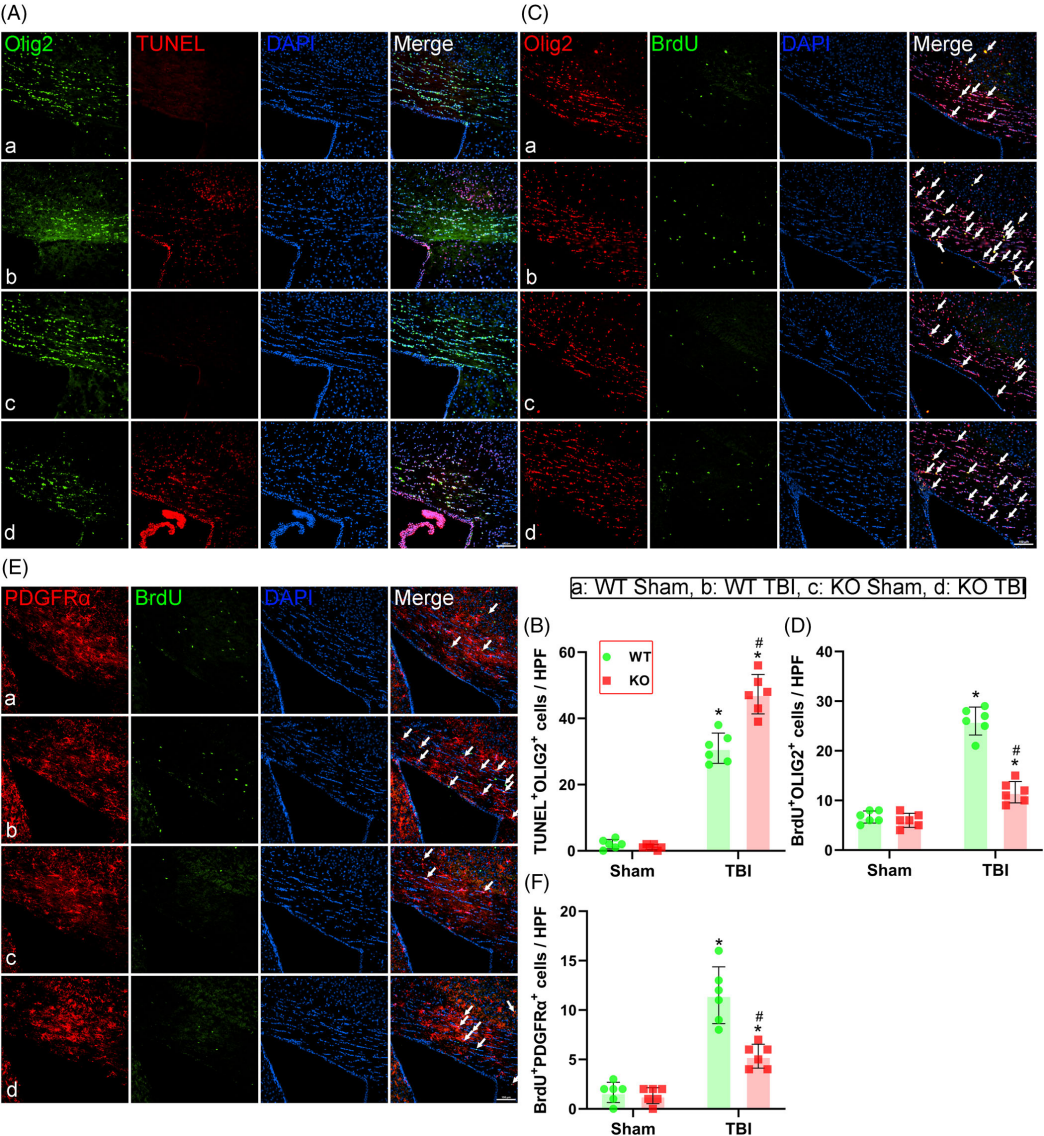
Triggering receptor‐2 (TREM2) KO promoted oligodendrocyte apoptosis and suppressed oligodendrocyte and oligodendrocyte precursor cell proliferation after traumatic brain injury (TBI). (A) Representative immunofluorescence images of the colocalization of oligodendrocytes (Olig2, green) with death marker (TUNEL, red) in corpus callosum area at 3 days after TBI. Nuclei were stained with DAPI (blue). Scale bar = 100 µm. (B) Quantitative analysis of the number of Olig2^+^TUNEL^+^ cells per high power field (HPF). (C) Representative images of the colocalization of Olig2 (Red) with proliferation marker (BrdU, Green) in corpus callosum area at 14 days after TBI. Nuclei were stained with DAPI (blue). Scale bar = 100 µm. (D) Quantitative analysis of the number of BrdU^+^Olig2^+^ cells per HPF. (E) Representative images of the colocalization of oligodendrocyte precursor cells (PDGFRα, Red) with BrdU (Green) in corpus callosum area at 14 days after TBI. Nuclei were stained with DAPI (blue). Scale bar = 100 µm. (F) Quantitative analysis of the number of BrdU^+^ PDGFRα^+^ cells per HPF. Groups allocation is shown in the black solid line box. ^*^ *p *< 0.05 versus WT Sham group, ^#^ *p *< 0.05 versus WT TBI group; *n* = 6 per group.

The genetic deletion of TREM2 aggravated white matter damage after TBI, as observed by the substantial myelin degradation in TREM2 KO mice. Oligodendrocytes wrap around axons and form an insulated structure, called the myelin sheath, that aids the efficient transmission of bioelectric signals, and maintains and protects normal neuron functioning. Moreover, oligodendrocyte precursor cells differentiate into oligodendrocytes in the CNS.^45^ Here, we observed a higher number of apoptotic oligodendrocytes in the WT TBI mice compared to the WT Sham mice at 3 days post‐TBI (*p* < 0.05, Figure 4A,B). Subsequently, to investigate whether early oligodendrocyte death leads to lasting changes in white matter repair, we quantified the replacement of oligodendrocytes and oligodendrocyte precursor cells post‐TBI by detecting BrdU incorporation into newly generated cells. Our results revealed that the number of both Olig2^+^BrdU^+^ and PDGFRα^+^BrdU^+^ cells was significantly higher in the WT TBI mice than in the WT Sham mice (*p* < 0.05, Figure 4C–F).

Notably, a significantly greater number of Olig2^+^TUNEL^+^ oligodendrocytes was observed in the corpus callosum of TREM2 KO mice 3 days after TBI compared to the WT TBI group (*p* < 0.05, Figure 4A,B). Additionally, we found a significant decrease in the number of Olig2^+^BrdU^+^ cells (*p* < 0.05, Figure 4C,D) and PDGFRα^+^BrdU^+^ cells (*p* < 0.05, Figure 4E,F) in the TREM2 KO TBI group compared to the WT TBI group at 14 days post‐TBI. However, without TBI, the TREM2 KO mice exhibited a normal oligodendrocyte status and oligodendrocyte precursor cell population, similar to that of the WT Sham mice.

### 3.5 TREM2 deficiency weakened the phagocytic activity of microglia after TBI

**FIGURE 5:**
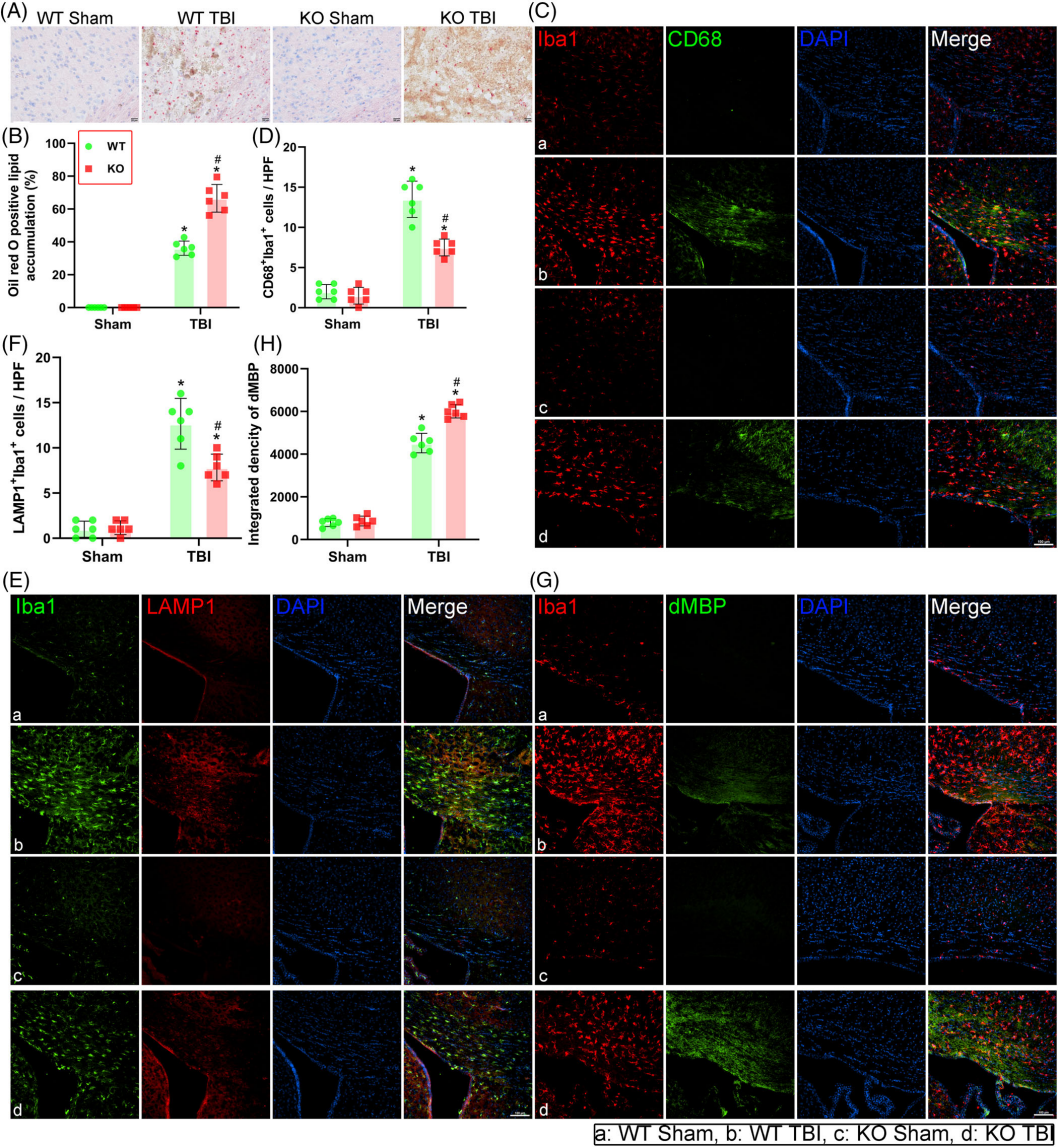
Triggering receptor‐2 (TREM2) deficiency weakened the phagocytic activity of microglia after traumatic brain injury (TBI). (A) Representative oil red O (ORO) staining images in corpus callosum area at 3 days after TBI. (B) Quantitative analysis of ORO positive area to the whole image in different groups. (C) Representative immunofluorescence images of the colocalization of microglia (Iba1, red) with CD68 (green) in corpus callosum area at 3 days after TBI. Nuclei were stained with DAPI (blue). Scale bar = 100 µm. (D) Quantitative analysis of the number of CD68^+^Iba1^+^ cells per high power field (HPF). (E) Representative immunofluorescence images of the colocalization of Iba1 (green) with LAMP1 (red) in corpus callosum area at 3 days after TBI. Nuclei were stained with DAPI (blue). Scale bar = 100 µm. (F) Quantitative analysis of the number of LAMP1^+^Iba1^+^ cells per HPF. (G) Representative immunofluorescence images of the colocalization of Iba1 (red) with dMBP (green) in corpus callosum area at 3 days after TBI. Nuclei were stained with DAPI (blue). Scale bar = 100 µm. (H) Quantitative analysis of the integrated dMBP immunofluorescence density in different groups. Groups allocation is shown in the black solid line box. ^*^ *p *< 0.05 versus WT Sham group, ^#^ *p *< 0.05 versus WT TBI group; *n* = 6 per group.

As mentioned before, microglial phagocytosis is critical for myelin repair in various CNS injuries. We next performed ORO staining and a series of immunofluorescence staining experiments to assess the phagocytic activity of microglia. Positive ORO staining is a hallmark of neutral lipid content and ORO staining is often performed on tissue from patients with atherosclerosis and demyelinated diseases such as experimental allergic encephalomyelitis and multiple sclerosis.^46^ Here, our ORO staining results revealed that the neutral lipids content was increased in WT TBI group compared with WT Sham group (*p *< 0.05, Figure 5A,B) and TREM2 deficiency further increased the neutral lipid content after TBI than that in the WT TBI mice (*p *< 0.05, Figure 5A,B). Next, to evaluate the activation states of microglia in the corpus callosum, we conducted double staining for Iba1 and CD68, which are considered microglial lysosomal and activated microglial markers.^47^ Results revealed that the number of Iba1^+^CD68^+^ cells was significantly greater in WT TBI group than that in WT Sham group, and the number of Iba1^+^CD68^+^ cells was lower in TREM2 KO TBI group than in WT TBI group (*p *< 0.05, Figure 5C,D), suggesting that TREM2 deficiency further disrupted the phagocytic activity of microglia after TBI. Furthermore, immunohistochemical studies have shown increased lysosomal‐associated membrane protein 1 (LAMP1) immunoreactivity in neurons, and in glial cells in Alzheimer's disease, indicating that LAMP1 is critical for the phagocytic activity of microglia.^48^ Next, we double stained for Iba1 and LAMP1 to further evaluate the phagocytic state of microglia after TBI. Our results demonstrated that the Iba1^+^LAMP1^+^ cells were significantly greater in WT TBI mice than in WT Sham mice, and the Iba1^+^LAMP1^+^ cells were lower in TREM2 KO TBI mice than in the WT TBI mice (*p *< 0.05, Figure 5E,F). Moreover, TREM2 KO TBI mice exhibited significant accumulation of myelin debris, as determined by the dMBP immunostaining results (*p *< 0.05, Figure 5G,H). These results revealed that deficient microglial phagocytosis induced by TREM2 depletion led to myelin clearance dysfunction. However, our results did not reveal any changes in microglial phagocytosis in mice subjected to TREM2 depletion without TBI stimulation.

### 3.6 TREM2 upregulation promoted white matter repair after TBI

**FIGURE 6:**
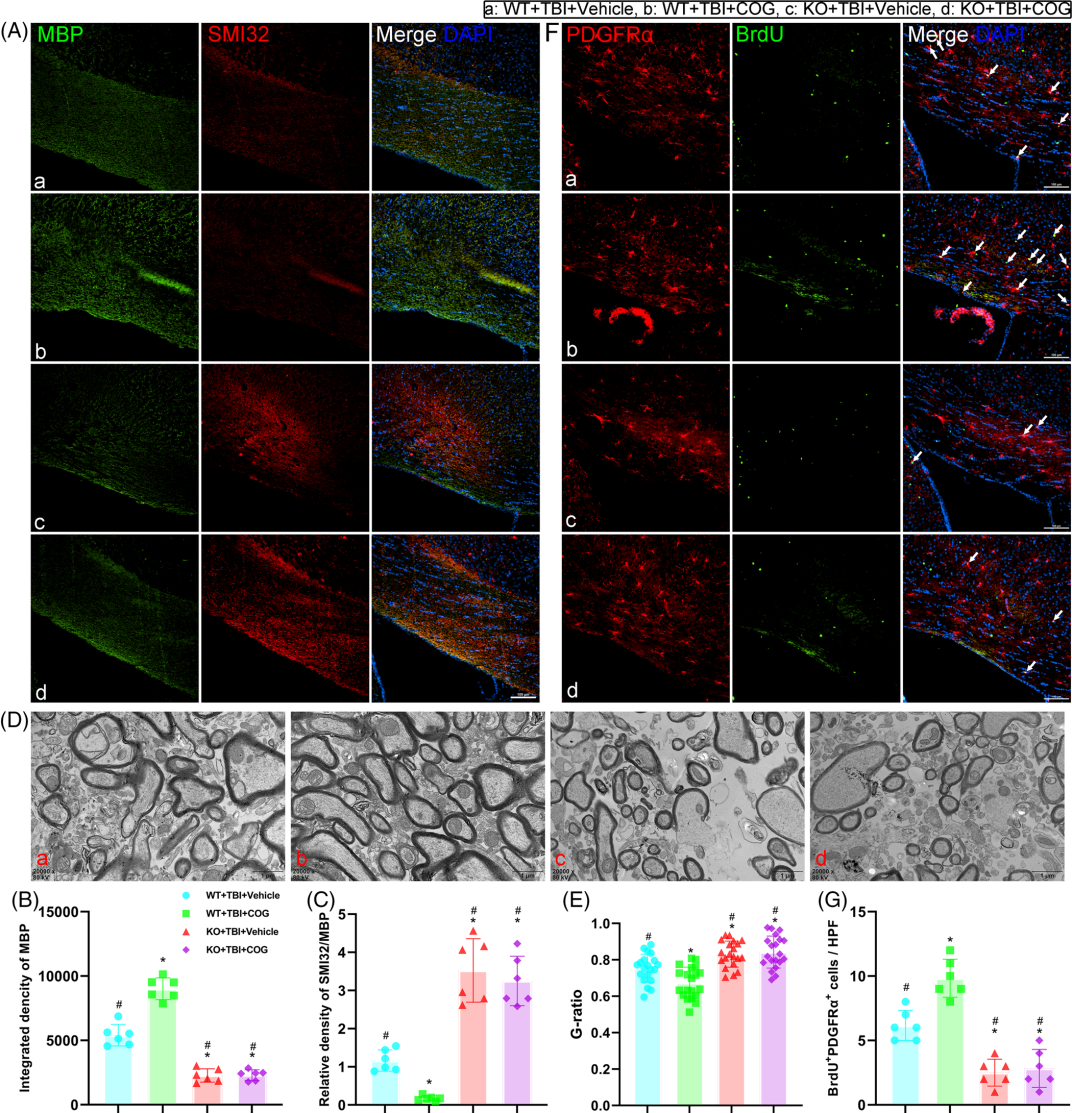
Triggering receptor‐2 (TREM2) upregulation promoted white matter repair after traumatic brain injury (TBI). (A) Representative immunofluorescence images of the colocalization of myelin basic protein (MBP) (green) with SMI32 (red) in corpus callosum area at 3 days after TBI. Nuclei were stained with DAPI (blue). Scale bar = 100 µm. (B and C) Quantitative analysis of the integrated density of MBP (B) and relative density of SMI32 to MBP (C). (D) Representative transmission electron microscopy (TEM) images of white matter tracts in corpus callosum region. (E) Quantitative analysis of the G‐ration in TEM experiments. (F) Representative immunofluorescence images of the colocalization of PDGFRα (red) with BrdU (green) in corpus callosum area at 14 days after TBI. Nuclei were stained with DAPI (blue). Scale bar = 100 µm. (G) Quantitative analysis of the number of the BrdU^+^ PDGFRα^+^ cells per high power field (HPF). Groups allocation is shown in the black solid line box. ^*^ *p *< 0.05 versus WT + TBI + vehicle group, ^#^ *p *< 0.05 versus WT + TBI + COG1410 group; *n* = 6 per group.

Given that genetic deletion of TREM2 exacerbated WMI after TBI, we next used the TREM2 agonist, COG1410, to increase TREM2 levels and further verify its effects on WMI after TBI. We found that TREM2 upregulation could alleviate WMI in WT mice after TBI, as evidenced by MBP intensity and the SMI32/MBP ratio (*p* < 0.05, Figure 6A–C). However, in the KO mice after TBI, COG1410 had no therapeutic effects to WMI (*p* > 0.05, Figure 6A–C). Similarly, by day 14 post‐TBI, TEM results indicated that COG1410 treatment mitigated myelin damage in WT mice after TBI (*p* < 0.05, Figure 6D,E). However, no therapeutic effects of COG1410 were found in the KO TBI mice (*p* > 0.05, Figure 6D,E). Additionally, we demonstrated that TREM2 upregulation could promote myelin remodelling in WT mice after TBI, as indicated by the greater number of PDGFRα^+^BrdU^+^ cells (*p* < 0.05, Figure 6F,G). However, TREM2 KO abolished theses effects (*p* > 0.05, Figure 6F,G). These findings indicate that TREM2 upregulation could alleviate WMI and promote remyelination after TBI, while TREM2 knockout exacerbates WMI and abolishes the protective effects of COG1410 after TBI.

### 3.7 TREM2 upregulation improved long‐term cognitive function recovery after TBI

**FIGURE 7:**
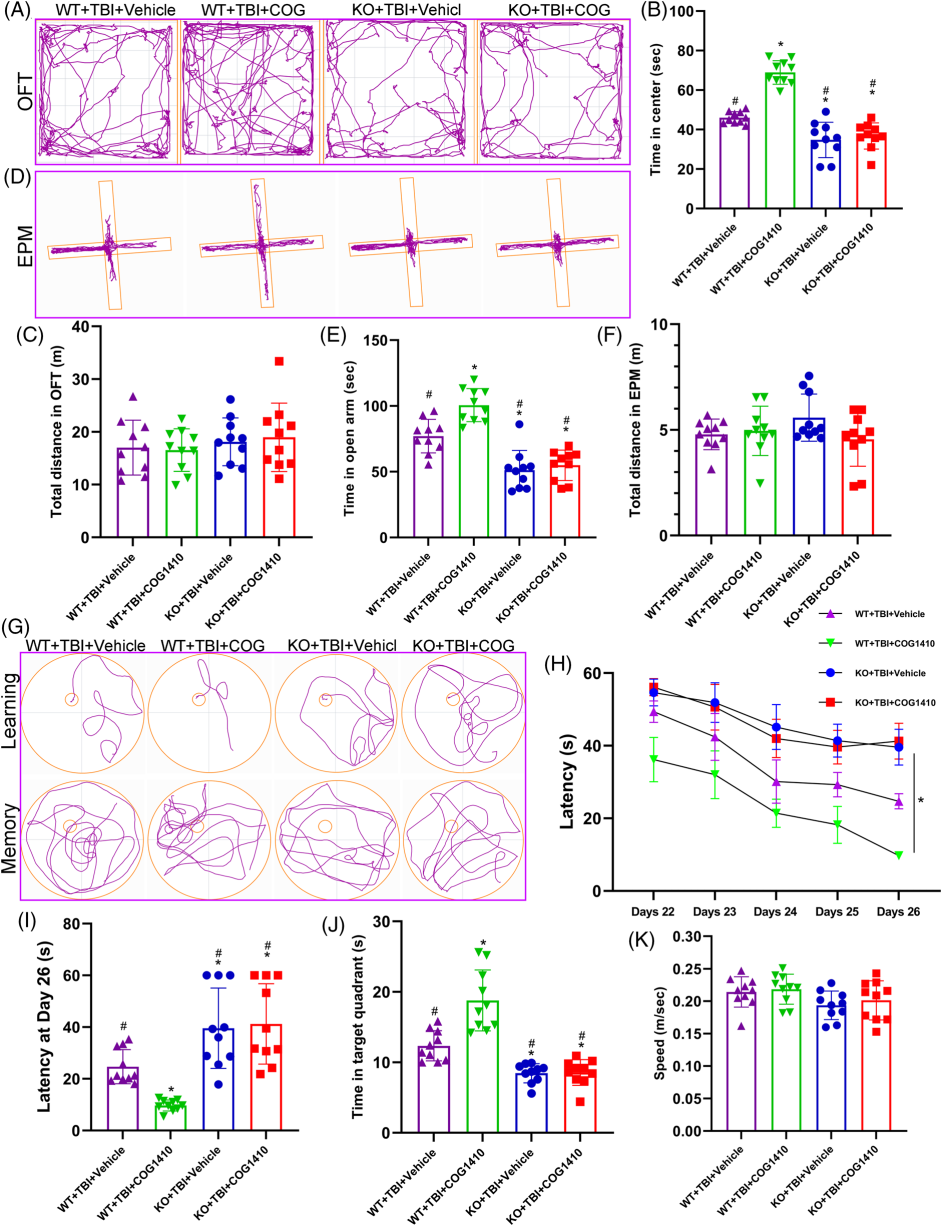
Triggering receptor‐2 (TREM2) upregulation improved long‐term cognitive function recovery after traumatic brain injury (TBI). (A and D) Representative images of the trajectory of mice in the open field test (OFT) (A) and elevated plus maze (EPM) (D) at 20 days after TBI. (B and C) Quantitative analysis of centre time (B) and total distance (C) travelled by mice in the OFT. (E and F) Quantitative analysis of open arms time (E) and total distance (F) travelled by mice in the EPM. (G) Representative images of the trajectory of mice in the Morris water maze (MWM). (H and K) Quantitative analysis of latency in the learning test (H and I), target quadrant time (J), and average swimming speed in the target quadrant test (K). ^*^ *p *< 0.05 versus WT + TBI + vehicle group, ^#^ *p *< 0.05 versus WT + TBI + COG1410 group; *n* = 10 per group. ^*^ *p *< 0.05 in H represents analysis of variance (ANOVA) results.

Since TREM2 upregulation could mitigate WMI after TBI, we wondered whether it could improve long‐term cognitive function recovery. The OFT, EPM test, and MWM test were performed again at 3 weeks after TBI. In the OFT and EPM, we found that TREM2 upregulation could alleviate anxiety‐like behaviours in WT TBI mice, as evidenced by more exploring time in the centre zone and open arms, respectively (*p* < 0.05, Figure 7A–F). TREM2 KO could impede these protective effects of COG1410 (*p* > 0.05, Figure 7A–F). Additionally, we reaffirmed that mice in all four experimental groups exhibited no motor deficits at this stage after TBI (*p* > 0.05, Figure 7C,F). Next, we demonstrated that TREM2 upregulation could improve learning and memory functions in WT mice after TBI by performing MWM test (*p* < 0.05, Figure 7G–J). In addition, TREM2 KO impeded these protective effects of COG1410 in KO mice after TBI (*p* > 0.05, Figure 7G–J). Additionally, mice in all four groups exhibited normal motor skills, and no differences in swimming speed were observed at the memory stage in the MWM test (*p* > 0.05, Figure 7K). Intriguingly, we demonstrated that TREM2 upregulation alleviated cognitive deficits at 3 weeks after TBI which could be mediated by a reduction in WMI, and we once again verified that TREM2 KO could exacerbate neurological behaviours after TBI.

### 3.8 Microglia were required for TREM2 to promote the proliferation of oligodendrocytes and oligodendrocyte precursor cells after TBI

**FIGURE 8:**
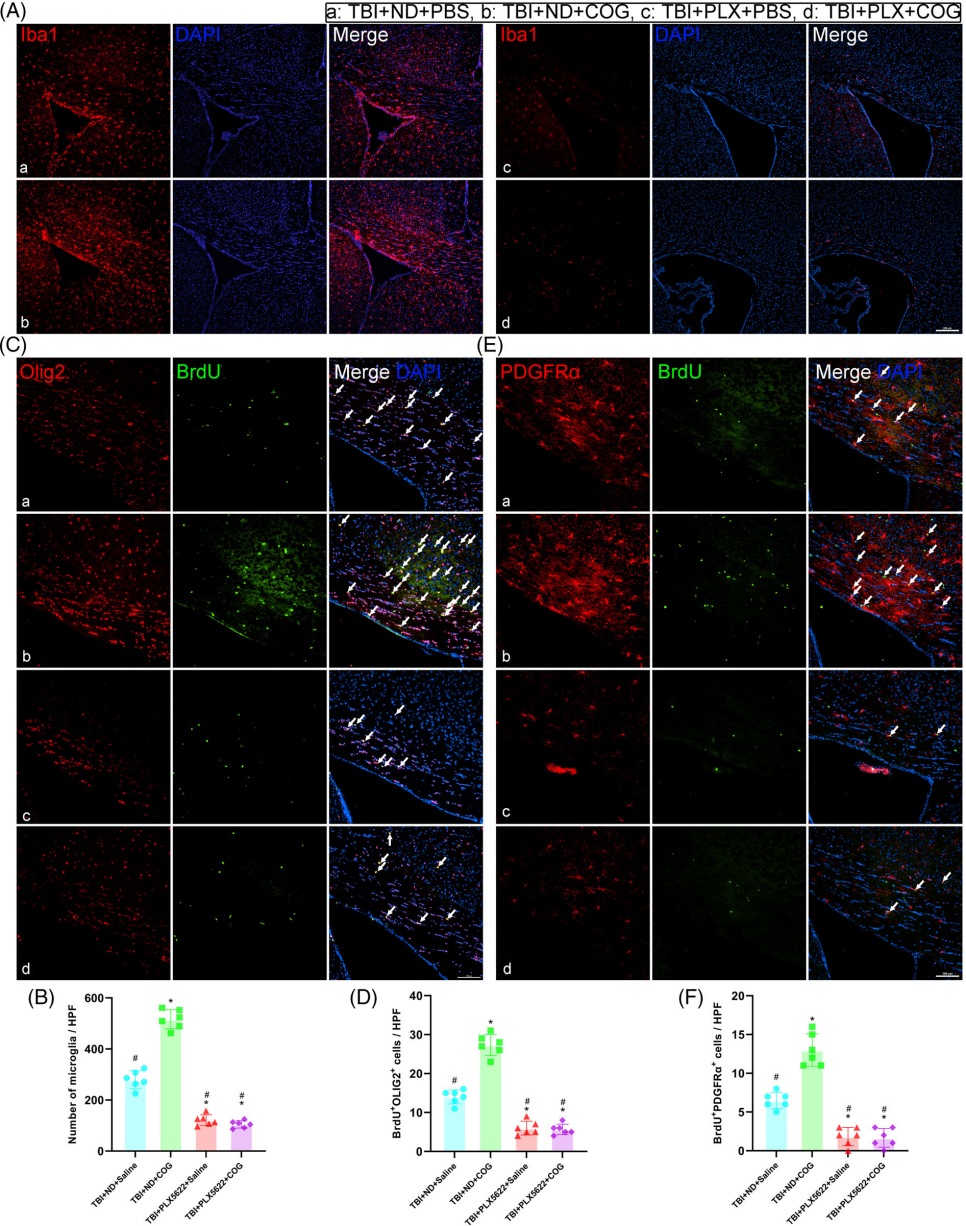
Microglia were required for triggering receptor‐2 (TREM2) to promote the proliferation of oligodendrocytes and oligodendrocyte precursor cells after traumatic brain injury (TBI). (A) Representative immunofluorescence images of the Iba1 (red) in corpus callosum area at 3 days after TBI. Nuclei were stained with DAPI (blue). Scale bar = 200 µm. (B) Quantitative analysis of the number of microglia per high power field (HPF) in different groups. (C) Representative immunofluorescence images of the colocalization of Olig2 (red) with BrdU (green) in corpus callosum area at 14 days after TBI. Nuclei were stained with DAPI (blue). Scale bar = 100 µm. (D) Quantitative analysis of the number of the BrdU^+^ Olig2^+^ cells per HPF. (E) Representative immunofluorescence images of the colocalization of PDGFRα (red) with BrdU (green) in corpus callosum area at 14 days after TBI. Nuclei were stained with DAPI (blue). Scale bar = 100 µm. (F) Quantitative analysis of the number of the BrdU^+^ PDGFRα^+^ cells per HPF. Groups allocation is shown in the black solid line box. ^*^ *p *< 0.05 versus TBI + ND + saline group, ^#^ *p *< 0.05 versus TBI + ND + COG1410 group; *n* = 6 per group.

As previously noted, our findings indicated that TREM2 impacted the proliferation of oligodendrocytes and oligodendrocyte precursor cells following TBI. These precursor cells were fundamental for remyelination processes post‐injury. We simultaneously treated WT TBI mice with COG1410 to upregulate TREM2 and PLX5622 to deplete microglia and explored the effects of both TREM2 upregulation and microglial depletion on the proliferation of oligodendrocytes and oligodendrocyte precursor cells. Consistent with the results of previous experiments, we found that the number of microglia in the corpus callosum was significantly greater in the TBI+ND+COG mice than in the TBI + ND + saline mice (*p *< 0.05, Figure 8A,B). We successfully depleted microglia via PLX5622 administration, and the number of microglia was dramatically lower in the two PLX5622‐treated mice than in the ND‐treated mice (*p *< 0.05, Figure 8A,B). Furthermore, we revealed significantly fewer Olig2^+^BrdU^+^ (*p *< 0.05, Figure 8C,D) and PDGFRα^+^BrdU^+^ cells (*p *< 0.05, Figure 8E,F) in PLX5622‐treated TBI mice than in ND‐treated mice 14 days after TBI, implying a dramatic reduction in the newly generated oligodendrocytes and oligodendrocyte precursor cells. Besides, our results revealed that TREM2 upregulation increased the newly generated oligodendrocytes and oligodendrocyte precursor cells without microglial depletion (*p *< 0.05, Figure 8C–F). Our results indicated that microglia were required for TREM2 to exert its effects after TBI.

### 3.9 TREM2 KO influenced microglial sterol metabolism‐related pathway in the white matter after TBI

**FIGURE 9:**
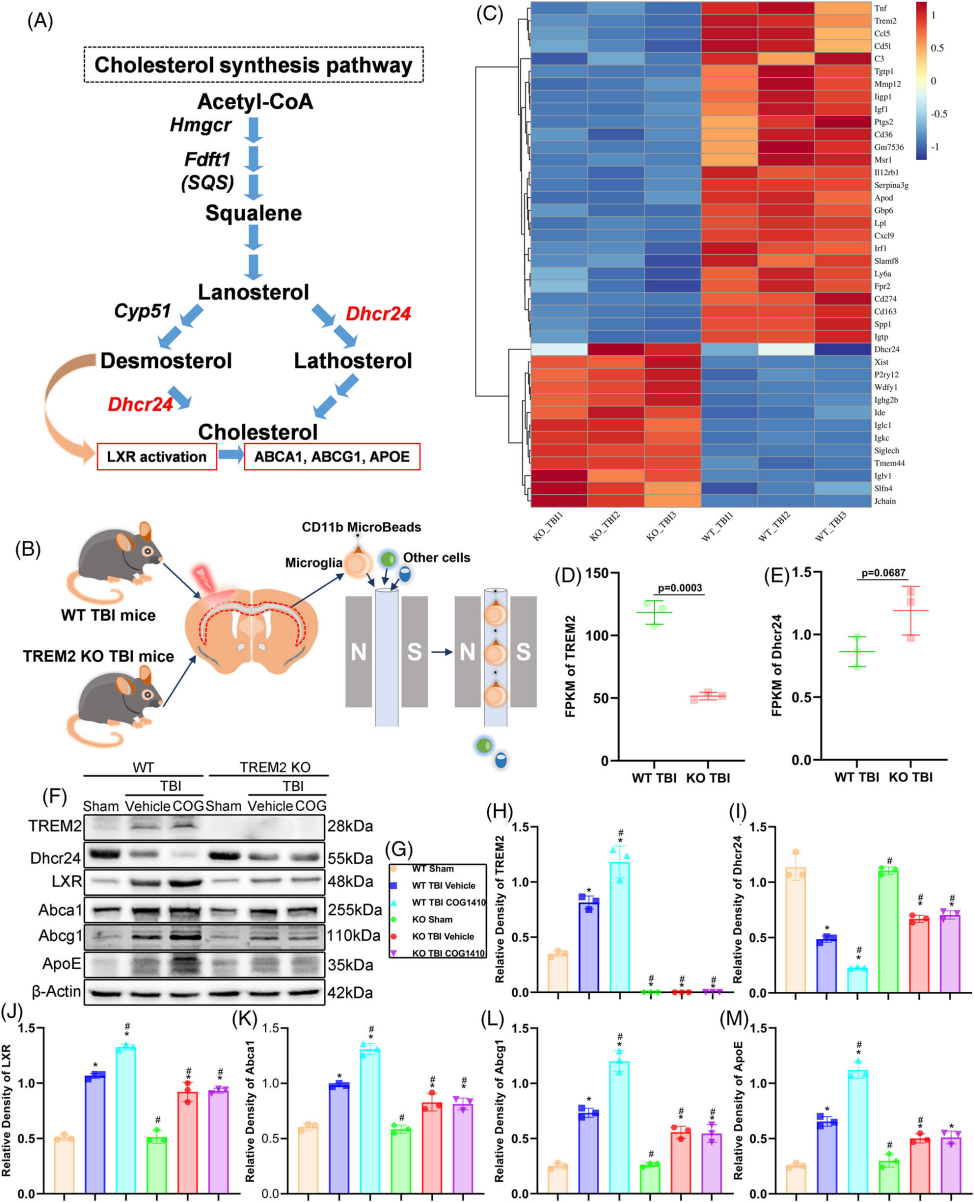
Triggering receptor‐2 (TREM2) KO influenced microglial sterol metabolism‐related pathway in the white matter after traumatic brain injury (TBI). (A) Cholesterol metabolism pathway according to the study by Berghoff et al. (B) Schematic diagram of sorting microglia in the corpus callosum of mice after TBI. (C) Heat map of differentially expressed genes that we were interested in. (D and E) We used fragments per kilobase of exon model per million mapped fragments (FPKM) to quantify the expression of TREM2 (D) and DHCR24 (E) between WT TBI mice and KO TBI mice. The *p* value is shown in the panel; *n* = 3 per group. (F) Representative western blot band of TREM2, DHCR24, LXR, Abca1, Abcg1, ApoE, and β‐actin by using the white matter tissues after TBI in different groups. (G) Allocation of different group. (H–M) Quantitative analysis of the expression of TREM2 (H), DHCR24 (I), LXR (J), Abca1 (K), Abcg1 (L), and ApoE (M) relative to β‐Actin. ^*^ *p *< 0.05 versus WT + Sham group, ^#^ *p *< 0.05 versus WT + TBI + vehicle group; *n* = 3 per group.

Taking together, these findings and the results of subsequent experiments suggest that TREM2 elevates the phagocytic ability of microglia and oligodendrocyte proliferation, thereby promoting white matter repair. However, the underlying mechanisms are poorly understood. A previous study demonstrated that in early remyelination after demyelinated diseases, microglia activate the LXR pathway to promote lipid transport and alleviate the neuroinflammatory environment by restricting cholesterol synthesis.^22^ In this study, 24‐dehydrocholesterol reductase (DHCR24) was downregulated and the synthesis of desmosterol, a LXR agonist, was elevated (Figure 9A).

In our study, microglia in the white matter at 3 days after TBI were sorted via a magnetic field (Figure 9B). Flow cytometry results showed that we extracted microglia with a purity of approximately 97% (Figure S2). Next, the isolated microglia were subjected to transcriptomics analysis, and the results demonstrated that the expression of various genes changed significantly in response to TREM2 depletion. We selected several TREM2 and sterol‐related genes for further analysis (Figure 9C). We found that the transcription level of TREM2 was significantly lower in the KO mice than in WT mice after TBI (*p *< 0.05, Figure 9C,D). Furthermore, we surprisingly found that the transcript level of DHCR24 tended to increase, while normally it is suppressed during normal remyelination, indicating that TREM2 KO truly disrupted the sterol metabolism in microglia (*p *= 0.067, Figure 9C,E). Next, we used white matter regions for western blotting, and analysed the changes in the expression of abnormal sterol metabolism‐related proteins after TBI. We found that TREM2 was upregulated after TBI in WT mice, and COG1410 treatment further enhanced the TREM2 expression than that in WT + TBI + vehicle mice (*p *< 0.05, Figure 9F,H).

In line with prior investigations of other demyelinating disorders, we observed a reduction in DHCR24 expression in the WT TBI mice, as compared to the WT Sham mice (*p *< 0.05, Figure 9F,I). Conversely, sham surgery did not elicit changes in DHCR24 expression between the two Sham groups (*p *> 0.05, Figure 9F,I). Notably, TREM2 upregulation via COG1410 in WT TBI mice further suppressed DHCR24 expression compared to the WT + TBI + vehicle mice (*p *< 0.05, Figure 9F,I). Additionally, DHCR24 was elevated in the KO TBI groups than in the WT + TBI + vehicle group but remained lower than in the two Sham groups, indicating that DHCR24 expression is not exclusively governed by TREM2 (*p *< 0.05, Figure 9F,I). Moreover, no discernible differences were found in DHCR24 expression between the two KO TBI group (*p *> 0.05, Figure 9F,I). Furthermore, we noted upregulation of LXR and its downstream targets Abca1, Abcg1, and ApoE in WT TBI mice than in WT Sham mice, with COG1410 administration further enhancing the expression of these proteins in the WT + TBI + COG mice than in the WT + TBI + vehicle mice (*p *< 0.05, Figure 9F, J–M). Moreover, LXR and its downstream proteins in the KO Sham group did not differ from that in the WT Sham mice (*p *> 0.05, Figure 9F, J–M). Nevertheless, TREM2 KO TBI group exhibited decreased expression of LXR and its downstream targets than in the WT + TBI + vehicle group (*p *< 0.05, Figure 9F, J–M), and no differences were found between the two KO TBI groups (*p *> 0.05, Figure 9F, J–M). Similarly, the expression of LXR and its downstream targets was still higher in the TBI groups than in the Sham groups (*p *< 0.05, Figure 9F, J–M). Our results implied that the body may initiate endogenous mechanisms akin to those involved in neurodegenerative diseases through early remyelination after TBI, partially regulated by TREM2.

### 3.10 LXR inhibition partially reversed the protective effects of TREM2 upregulation

**FIGURE 10:**
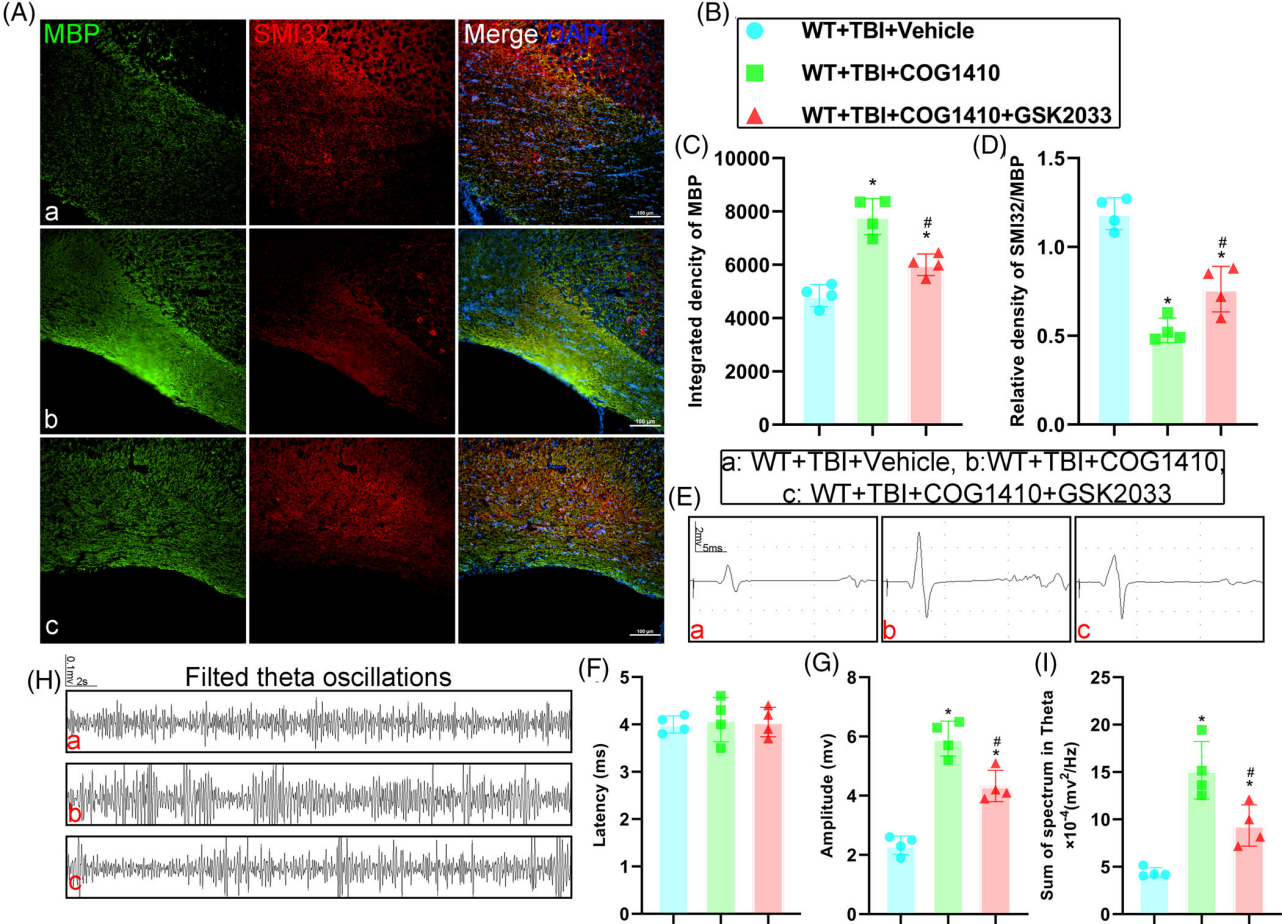
LXR inhibition partially reversed the protective effects of triggering receptor‐2 (TREM2) upregulation. (A) Representative immunofluorescence images of the colocalization of myelin basic protein (MBP) (green) with SMI32 (red) in corpus callosum area at 3 days after traumatic brain injury (TBI). Nuclei were stained with DAPI (blue). Scale bar = 100 µm. (B) Allocation of different group in C and D. (C and D) Quantitative analysis of the integrated density of MBP (C) and relative density of SMI32 to MBP (D). (E) Representative images of motor evoked potentials (MEPs) in different groups. Scale bar = 2 mv or 5 ms, respectively. (F and G) Quantitative analysis of the latency (F) and the amplitude (G) of the MEPs in different groups. (H) Representative images of local field potentials in CA1 of different groups. Scale bar = 0.1 mv or 2 s, respectively. (I) Quantitative analysis of the sum of the spectrum in theta oscillations in different groups. Groups allocation is shown in the black solid line box. ^*^ *p *< 0.05 versus WT + TBI + vehicle group, ^#^ *p *< 0.05 versus WT + TBI + COG1410 group; *n* = 4 per group.

LXR is the core target of special sterol metabolism in early myelin remodelling. To further explore the relationship between TREM2 and sterol metabolism, GSK2033 was used to inhibit LXR. Consistent with our previous findings, TREM2 demonstrated neuroprotective effects by improving white matter integrity, as evidenced by increased MBP fluorescence intensity and decreased SMI32/MBP ratio (*p *< 0.05, Figure 10A–D). However, inhibition of LXR partially attenuated these effects in the WT+TBI+COG+GSK mice compared to the WT+TBI+COG mice (*p *> 0.05, Figure 10A–D). Subsequently, we conducted in vivo electrophysiological recordings 14 days post‐TBI in these three groups. Analysis of MEP recordings revealed no significant differences in latency in these three groups (*p *> 0.05, Figure 10E,F). Nevertheless, TREM2 activation increased the amplitude of MEPs in COG‐treatment but no LXR inhibitor mice compared with the vehicle mice (*p *< 0.05, Figure 10E,G). Conversely, inhibition of LXR significantly decreased the amplitude of MEPs compared to the COG‐treatment but no LXR inhibitor mice, although it remained higher than in the vehicle group (*p *< 0.05, Figure 10E,G). Moreover, COG1410 treatment enhanced theta LFP activity in the CA1 region compared to vehicle treatment (*p *< 0.05, Figure 10H,I). Nevertheless, LXR inhibition partially reversed these effects (*p *> 0.05, Figure 10H,I). Our results indicated that TREM2‐mediated neuroprotection involves the activation of the LXR pathway, contributing to improved white matter integrity and electrophysiological activities following TBI.

## 4 DISCUSSION

Excessive external force applied to the brain causes TBI, which is a significant global public health concern. Although motor deficits tend to improve with time, cognitive impairments can persist, leading to substantial economic and social burdens.^49^ So far, numerous studies have concentrated on cortical grey matter, with limited exploration into the involvement of white matter after TBI. Moreover, no clinical therapies for WMI after TBI have been successful thus far. Most preclinical studies related to WMI after TBI have focused on alleviating the neuroinflammatory response; indeed, they observed beneficial effects, and microglia were found to be indispensable in this process. One study showed that specifically deleting histone deacetylase 3 in microglia mitigated neuroinflammation and enhanced white matter integrity in a mouse model of TBI.^50^ In addition, neuroprotective medications such as ethyl pyruvate improved white matter remodelling by promoting microglial state conversion in a rat TBI model.^51^ Other studies have demonstrated that alleviating endoplasmic reticulum stress and blood–brain barrier damage reduces neuroinflammation and eventually improves white matter integrity.^52^, ^53^ Furthermore, other studies revealed that microglia alleviate WMI by interacting with oligodendrocytes.^54^, ^55^ However, the exact underlying mechanisms have yet to be elucidated.

TREM2 serves as a receptor that interacts with a diverse array of ligands, several of which are associated with tissue damage, such as myelin debris following demyelination post‐TBI.^25^ The significance of TREM2 in human health was initially recognized in Nasu‐Hakola disease by scientists, which involves pathological changes in whiter matter.^26^ In recent years, researchers have underscored the significance of TREM2 in myeloid cells as a pivotal immune signalling hub induced by pathology. Moreover, mounting evidence suggests that TREM2 plays beneficial roles in diverse CNS diseases. In Alzheimer's disease, a reduction in TREM2 results in diminished microglial activity, triggering neuroinflammation and ultimately accelerating the aging process and neural loss. More precisely, the absence of TREM2 inhibits microglial survival and impedes the generation of disease‐associated microglia, which can restrict plaque expansion by forming a barrier around Aβ. These microglia are distinguished by their heightened phagocytic activity and participation in lipid metabolism.^56^ However, the relationship between TREM2 and Alzheimer's disease is complicated, and there is still no consensus on the role of TREM2 in Alzheimer's disease. However, it is generally agreed upon that TREM2 activation can have both beneficial and negative effects, and that the timing of TREM2 activation relation to disease stage is important. A previous investigation associated with experimental multiple sclerosis demonstrated that upregulation of TREM2 in bone marrow‐derived myeloid precursor cells augmented lysosomal and phagocytic activity. This resulted in the clearance of degenerated myelin and a decrease in the inflammatory response.^57^ Likewise, a deficiency in TREM2 exacerbates neurodegeneration and neuroinflammation induced by α‐synuclein in an experimental Parkinson's disease study.^58^ In addition, TREM2 has been shown to play protective roles against other CNS injuries, such as intracerebral haemorrhage, subarachnoid hemorrhage, ischemic stroke, and TBI.^29^ Most of these studies focused on neuroinflammation and neural body loss under conditions of TREM2 upregulation or KO; however, the effects of TREM2 on white matter after injury have been ignored. In our previous study, TREM2 expression peaked at 3 days at injuries site after TBI in mice. Additionally, except microglia, TREM2 was also expressed on neurons at the peri‐injury site.^29^ Here, we also found that TREM2 expression in the white matter peaked at 3 days, indicating that endogenous TREM2 upregulation is involved in defence against injuries. In addition, we revealed that TREM2 expressed mainly on microglia. Furthermore, a previous study demonstrated that TREM2 is expressed on astrocytes, neurons, and microglia.^59^ We speculated that soluble TREM2 (sTREM2) might be involved in this phenomenon. In humans, TREM2 can be cleaved by α‐secretases disintegrin, metalloproteinase domain‐containing protein 17 (ADAM17), and ADAM10, subsequently generating sTREM2.^60^ Furthermore, astrocytes are rich in white matter and play a role in both physiological and pathological conditions.^61^ We speculated that activated microglia release sTREM2 into the microenvironment that subsequently attaches to astrocytes and neurons.^59^ However, this phenomenon needs to be further explored. In our study, we found that most microglia in the white matter expressed TREM2 after TBI. Moreover, TREM2 depletion aggregated long‐term cognitive behavioural abnormalities after TBI, which were aligned with the results from our previous study.^29^ Cognitive deficits are very common among patients with TBI. Most TBI patients exhibit long‐term memory deficits, mood disorders, and fatigue, although mortality has declined over the past few years.^62^ Additionally, in an animal TBI model, mice displayed enduring deficits in learning and memory, along with the manifestation of psychiatric disorders.^44^ In our present study, we found that mice showed anxiety‐like behaviour and learning and memory deficits at 3 weeks after TBI. In line with the results of our earlier study, TREM2 KO did not negatively affect neurological functions under physiological conditions; however, TREM2 depletion aggregated long‐term cognitive behavioural abnormalities during TBI challenge. Additionally, our findings indicated that increasing TREM2 expression could mitigate long‐term cognitive deficits, while depletion of TREM2 nullified the protective effects of COG1410, consistent with our earlier observations. In our previous report, we highlighted TREM2's role in mitigating neuroinflammation and neuronal loss at sites of injury following TBI, yet the significance of white matter involvement was ignored.^29^

Here, we found that mice exhibited significant WMI after TBI, which was consistent with the findings of other studies,^34^, ^44^ and manifested as white matter structural integrity damage of, MBP loss, increased SMI32/MBP ratio, and NOR destruction early after TBI. At 14 days after TBI, we further demonstrated a reduction in white matter via DTI and a decrease in myelin content via TEM. We demonstrated that TREM2 KO did not influence the physiological function of white matter; however, TREM2 KO dramatically exacerbated WMI after TBI induction. Remyelination relies on novel, mature oligodendrocytes, which differentiate from oligodendrocyte precursor cells.^45^ Our current study revealed that TREM2 KO enhanced the apoptosis of mature oligodendrocytes while inhibiting the proliferation of new oligodendrocytes. Furthermore, TREM2 depletion hindered the proliferation of oligodendrocyte precursor cells. These results suggested that in addition to influencing grey matter, TREM2 also affects white matter after TBI. Indeed, a previous study reported that in patients with small vessel disease, plasma sTREM2 showed a significant association with the volume of white matter hyperintensity. In addition, in patients with Alzheimer's disease or cerebral amyloid angiopathy, sTREM2 also showed a significant association with the volume of white matter hyperintensity.^63^ In our present study, we also revealed that TREM2 upregulation by COG1410 could alleviate WMI and promote the proliferation of oligodendrocyte precursor cells. These results suggested that TREM2 in the CNS might be related to white matter pathology after TBI.

As mentioned before, microglia can promote remyelination in the CNS after injury via clearance of debris and modulation of the extracellular microenvironment and TREM2 is critical for the survival and phagocytosis of microglia. A previous study reported that the TREM2‐mediated microglial response to oligodendrocyte differentiation and remyelination could be promoted by physical exercise in rats after ischemia stroke and that ischemic stroke was also associated with abundant WMI pathology.^64^ In a TBI‐induced tauopathy mouse model, TREM2 deficiency increased microglial accumulation in the corpus callosum in the chronic phase, which resulted in exacerbated WMI.^65^ In addition, TREM2 KO microglia exhibits defects in their ability to engulf myelin debris. In a spinal cord demyelination model, TREM2 restored microglial responsiveness and improved lipid clearance by phagocytes.^66^, ^67^ On the flip side, TREM2 deficiency diminishes the phagocytosis of harmful products of organism.^25^ In our study, we demonstrated that TREM2 KO decreased the phagocytic ability of microglia in the corpus callosum, as evidenced by an elevated neutral lipid content and a reduced number of activated and phagocytic microglia, which eventually resulted in dMBP accumulation. A previous study indicated that TREM2 KO microglia exhibits defects in the migration and phagocytosis of myelin debris.^66^ Nevertheless, Nugent et al. reported that in a cuprizone‐induced demyelination model, microglia deficient in TREM2 could phagocytose myelin debris but struggled to eliminate intracellular cholesterol.^28^ In our study, we found that TREM2 KO suppressed the activation of microglia and reduced the number of microglia in the corpus callosum. Moreover, we found that the phagocytic activity of microglia was decreased in the corpus callosum of TREM2 KO TBI mice. Considering that the number of microglia was decreased, we hypothesized that the overall phagocytic function of microglia was reduced. Indeed, a decrease in the overall phagocytic capacity of microglia could have adverse effects on WMI after TBI. This also explains why studies that regulate the state of microglia have shown improvements in WMI after various diseases. Moreover, through the upregulation of TREM2 by COG1410, we found that WMI could be suppressed in WT mice but not in KO mice after TBI. Furthermore, COG1410 improved long‐term cognitive deficits; however, TREM2 KO abolished these protective effects. As mentioned earlier, successful remyelination requires oligodendrocyte production and oligodendrocyte precursor cell proliferation. To verify that TREM2 regulated remyelination by acting through microglia, we treated mice with microglial inhibitor PLX5622 before and after TBI. In PLX5622‐treated mice, microglia were successfully depleted, and the proliferation of oligodendrocytes and oligodendrocyte precursor cells was suppressed, indicating that TREM2 regulates remyelination by acting through microglia. Our results suggested that TREM2 could mitigate WMI after TBI by regulating microglial phagocytosis and subsequent oligodendrocyte and oligodendrocyte precursor cell proliferation. The anti‐neuroinflammatory role of TREM2 might partially explain these effects.^29^

While the crucial phagocytic role of microglia in remyelination is widely acknowledged, the metabolic pathways necessary for the clearance of myelin debris remain poorly understood. Recently, Berghoff et al. reported that repair of acutely demyelinated lesions in demyelinating experimental models requires sterol synthesis in microglia. Intriguingly, rather than producing cholesterol, desmosterol synthesis, which is the immediate cholesterol precursor, was the determinant desmosterol activated LXR signalling to alleviate neuroinflammation and produce a favourable environment for remyelination. Additionally, products of LXR target genes aided in the efflux of lipids and cholesterol from lipid‐laden microglia, contributing to the support of remyelination by oligodendrocytes.^22^ These excellent findings provoked our extreme interest in whether this process occurs early after TBI and whether TREM2 exerts its effects on this biological process. We speculated that, like those in neurodegenerative diseases, microglia absorb abundant cholesterol from myelin debris after TBI. Although excessive cholesterol inhibits cholesterol synthesis via a feedback mechanism, the proinflammatory environment early after TBI promotes cholesterol synthesis.^68^, ^69^ Berghoff et al. reported that, early remyelination, the transcript levels of sterol‐synthesizing enzymes, except DHCR24, were upregulated in microglia.^22^ DHCR24 is an enzyme that transforms lanosterol to lathosterol and desmosterol to cholesterol. Nuclear factor erythroid 2 related factor‐1 (Nrf1) is an inflammation‐responsive transcriptional repressor on the endoplasmic reticulum that can bind to cholesterol and subsequently suppress inflammation and promote cholesterol excretion.^70^ Nrf1 can mediate DHCR24 expression in the early stage of neurodegenerative diseases.^22^ Then, we extracted corpus callosum tissue from WT and KO mice at 3 days after TBI, sorted microglia, and performed a transcriptomic analysis. Surprisingly, DHCR24 was upregulated in TREM2 KO mice, whereas DHCR24 was downregulated during early demyelination processes under physiological conditions. Unfortunately, we could not perform western blot analysis using sorted microglia, because the amount of protein extracted was not sufficient to complete the western blot analysis. As a result, we extracted white matter tissue samples to analyse sterol‐related proteins via western blotting. We found that TREM2 upregulation in WT TBI mice further impeded the expression of DHCR24, and LXR and its target proteins changed accordingly. However, TREM2 KO increased the expression of DHCR24, and the LXR and its target proteins also changed accordingly. A previous study indicated that stimulation with LXR could promote the expression of TREM2.^71^ TREM2 is recognized as a crucial transcriptional regulator of cholesterol transport and metabolism during prolonged myelin phagocytic activity. TREM2 deficiency leads to pathological lipid accumulation in microglia, which can be rescued by LXR agonists.^28^ In addition to promoting cholesterol elimination in microglia, LXR signalling can alleviate neuroinflammation and produce a favourable environment for remyelination in demyelinating disease.^22^ In addition, the anti‐inflammatory effects of TREM2 are widely accepted.^25^ Furthermore, our group and others have shown that TREM2 promotes phagocytic activity of microglia and a previous study showed that the uptake of myelin skews microglia toward an immunosuppressive and neurotrophic phenotype.^24^, ^72^ Our results revealed that the interactions among TREM2, DHCR24, and LXR are complex and need to be explored in detail. Our findings may lead to the identification of another promising target for intervention in WMI after TBI. Finally, we used GSK2033 to inhibit LXR, while TREM2 was upregulated by COG1410 simultaneously. We found that LXR inhibition could partially reverse the effects of TREM2 upregulation by increasing WMI and suppressing electrophysiological activity after TBI.

**FIGURE 11:**
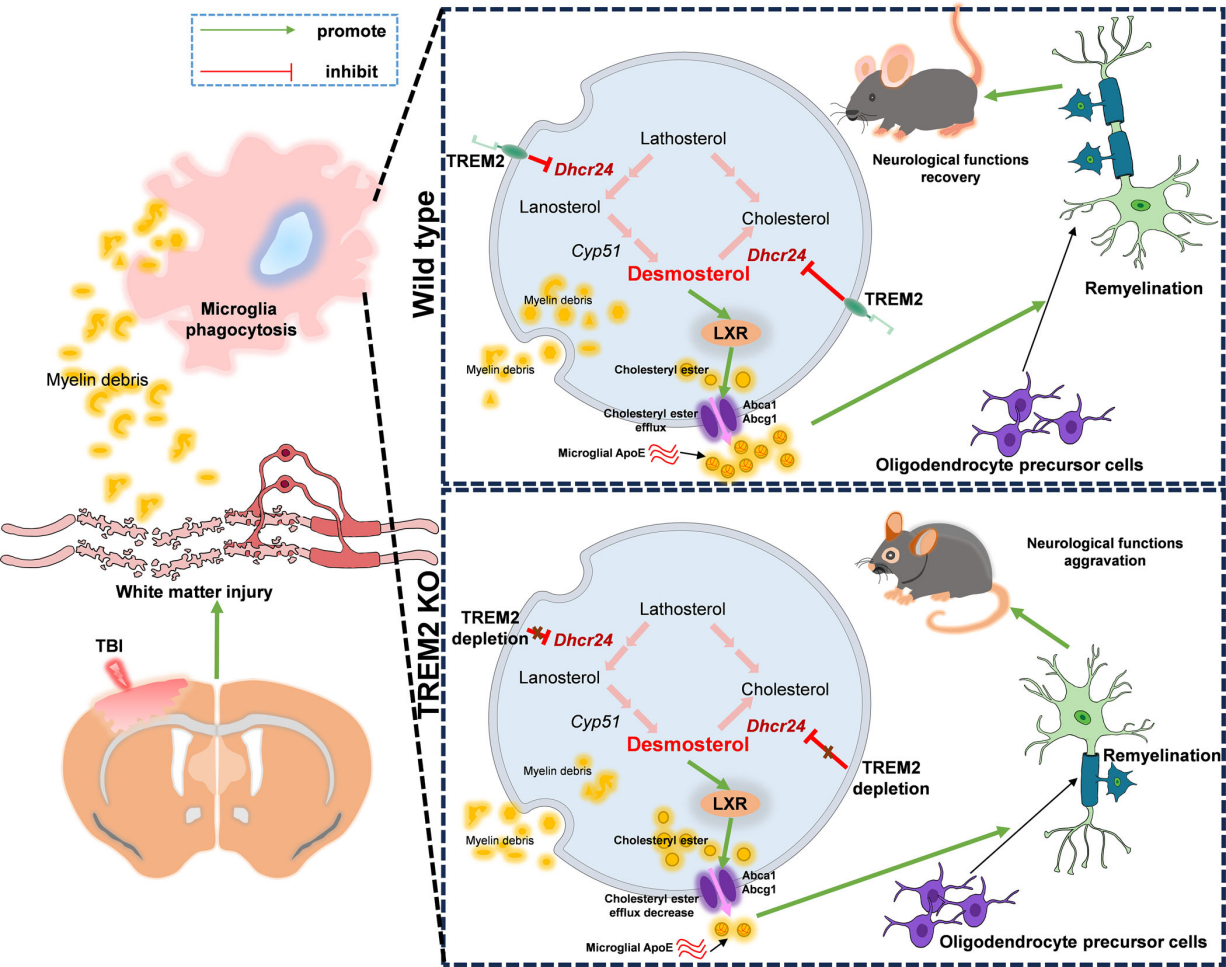
Schematic summary of present study. Our results demonstrated that depletion of triggering receptor‐2 (TREM2) exacerbated white matter injury (WMI) after traumatic brain injury (TBI) by suppressing holistic microglial phagocytosis and caused augment of oligodendrocytes apoptosis and abatement of oligodendrocyte precursor cells proliferation, which might be mediated by microglial sterol metabolism. On the contrary, upregulation of TREM2‐ameliorated WMI might be mediated by regulating DHCR24/LXR pathway in microglia. TREM2 might promote cholesterol ester efflux and the recycled cholesterol was reused to accelerate oligodendrocytes reproduction from oligodendrocyte precursor cells proliferation, thereupon then, facilitating neurological functions recovery after TBI. However, these complicated interacts between TREM2 and WMI should be further explored.

Taken together, our results demonstrated that TREM2 could regulate WMI after TBI in mice (Figure 11). In brief, abolition of TREM2 exacerbates WMI after TBI by suppressing microglial phagocytosis, augmenting oligodendrocyte apoptosis, and decreasing oligodendrocyte precursor cell proliferation, which might be mediated by microglial sterol metabolism. In contrast, the amelioration of WMI by TREM2 upregulation might be mediated by the regulation of microglial sterol metabolism through the TREM2/DHCR24/LXR pathway. TREM2 might promote cholesterol ester efflux and recycled cholesterol can be used to accelerate oligodendrocyte differentiation from oligodendrocyte precursor cells, facilitating neurological function recovery after TBI. However, these complicated interactions between TREM2 and WMI should be further explored. Moreover, TBI can cause blood–brain barrier injury that triggers local neuroinflammation and recruitment of peripheral immune cells.^73^ Consequently, both infiltrating macrophages and microglia are involved in TBI. In addition, PLX5622 affects both peripheral and central immune cells.^74^ As a result, we were unable to exclude the influence of macrophages in this study. More experiments should be conducted to explore these ambiguities. Overall, we demonstrated that the alleviation of WMI after TBI in mice by TREM2 might be mediated by the regulation of DHCR24/LXR pathway in microglia.

## AUTHOR CONTRIBUTIONS

Zhao Li: Conceptualization; investigation; data curation; writing—original draft; funding acquisition. Shenghui Yu: Data curation; validation; writing—original draft. Lin Li: Formal analysis; writing—review and editing. Chao Zhou: Methodology. Lin Wang: Writing—review and editing. Shuang Tang: Software. Nina Gu: Methodology. Zhaosi Zhang: Supervision. Zhijian Huang and Hong Chen: Methodology; funding acquisition. Wei Tang and Yingwen Wang: Validation. Xiaomin Yang: Project administration; supervision; writing—review and editing. Xiaochuan Sun: Project administration; supervision; writing—review and editing; funding acquisition. Jin Yan: Conceptualization; investigation; data curation; writing—original draft; project administration; supervision; methodology; software; writing—review and editing.

## CONFLICT OF INTEREST STATEMENT

The authors declare no conflicts of interest.

## FUNDING INFORMATION

Sichuan Province Medical Youth Innovation Research Project Plan, Grant No.: Q23087; National Natural Science Foundation of China, Grant No.: 82102316; Chongqing Doctor's ‘Direct Train' Research Project, No. CSTB2022BSXM‐JCX0042; National Natural Science Foundation of China, Grant No.： 82071397.

## ETHICS STATEMENT

All procedures were approved by the Ethics Committee of Chongqing Medical University and carried out in accordance with ARRIVE guidelines and the National Institutes of Health Guide for the Care and Use of Laboratory Animals.

## Supporting information

Supporting Information

Supporting Information

## Data Availability

The authors declare that all supporting data and materials are available within the article and the raw data can be obtained under reasonable requirements from the corresponding author.
