# Supplementary material for: TREM2 alleviates white matter injury after traumatic brain injury in mice might be mediated by regulation of DHCR24/LXR pathway in microglia
Source: Clin Transl Med. 2024 Apr 22;14(4):e1665. doi: 10.1002/ctm2.1665 (PMC11035381; doi:10.1002/ctm2.1665)
Supplement: Supplementary file 1 — Supporting Information [file CTM2-14-e1665-s001.docx]

**Supplemental Materials**

**TREM2 alleviates white matter injury after traumatic brain injury in mice might be mediated by regulation of DHCR24/LXR pathway in microglia**

**Supplemental methods**

**Supplemental Figures and Figure Legends**

**Supplemental methods**

**TREM2 genotype identification by polymerase chain reaction (PCR)**

We purchased four male and four female homozygote TREM2 KO mice from Jackson Lab as F0 generations. We used PCR to confirm the TREM2 genotype of the these F0 generations. Briefly speaking, tails of these eight mice and another wildtype (WT) mouse were collected to analysis. The WT mouse was used to as control in TREM2 genotype identification. Total DNA was extracted from tails of mice using an Animal Genomic DNA Quick Extraction Kit for PCR Analysis (Cat# D0065S, Beyotime Biotechnology, Shanghai, China) according to the manufacturer’s instruction. PCR systems were set up in 20 µl volumes and PCR was performed on a PCR System (Bio-Rad, USA). The 20 µl volumes PCR systems included 8 µl ddH_2_O, 10 µl 2 × Taq Master Mix (Cat# P112-AA, Vazyme), 0.5 µl forward primer (10 pmol/µl), 0.5 µl reverse primer (10 pmol/µl), and 1 µl genomic DNA. The program included 5 min at 95 °C; 10 cycles of 30 s at 95 °C ,30 s at 60 °C, and 1 kb/min at 68 °C; 26 cycles of 30 s at 95 °C ,30 s at 55 °C, and 1 kb/min at 72 °C; 5 min at 72 °C; 2 min at 10 °C. Then, the concentration of agarose gel was 2% (Cat# 1110GR100, BioFroxx) in agarose gel electrophoresis. The DNA was dyed by nucleic acid dye (GelstainRedTM, Cat# S2009L, UE). Quantity of base pair was instructed by DNA marker (Cat# BM411-02, Trans DNA Marker Ⅱ). The sequences of the primers for TREM2 mutant genotype as follows: forward sequence, 5’-TTACACAAGACTGGAGCCCTGAGGA-3’; reverse sequence, 5’-TCTGACCACAGGTGTTCCCG-3’. The sequences of the primers for WT genotype as follows: forward sequence, 5’-CCCTAGGAATTCCTGGATTCTCCC-3’; reverse sequence, 5’-TCTGACCACAGGTGTTCCCG-3’. Results are shown in Suppl. Fig. 1.

**Microglial purity by flow cytometry**

The purity of our sorted microglia was verified by flow cytometry. We used a naïve mouse to sort the microglia of the whole brain tissue by the methods mentioned in the materials and methods part of the text. Then, the sorted CD11b^+^ cells were added with FITC-labeled anti-CD11B monoclonal antibody and APC-labeled anti-CD45 monoclonal antibody, and were incubated for 30min under dark condition and washed. Finally, the purity of CD11b^+^CD45^+^ cells were detected by flow cytometry and the results is shown in Suppl. Fig. 2.

**The primary antibodies in Western blot**

Anti-β-actin, Abcam ab213262, 1:5000; anti-TREM2, Cell Signaling Technology 91068, 1:1000; anti-Dhcr24, ABclonal A5402, 1:1000; anti-LXR, ABclonal A3974, 1:1000; anti-Abca1, ABclonal A22125, 1:1000; anti-Abcg1 ABclonal A17907, 1:1000; and anti-ApoE, ABclonal A16344, 1:1000.

**The primary antibodies in immunofluorescence staining**

Anti-Iba1, Abcam ab5076, 1:200; anti-TREM2 Cell Signaling Technology 91068, 1:400; anti-MBP, Sigma-Aldrich MAB386, 1:400; anti-SMI32, BioLegend 801701, 1:400; anti-Nav1.6, Alomone ASC-009, 1:300; anti-Caspr, Santa Cruz sc-373777, 1:200; anti-Olig2, Sigma-Aldrich MABN50, 1:400; anti-BrdU, Abcam ab6326, 1:250; anti-PDGFRα, Abcam ab203491, 1:500; anti-dMBP, Sigma-Aldrich AB5864, 1:400; anti-CD68, Abcam ab125212, 1:400; and anti-LAMP1, Abcam ab25245, 1:400.

**Supplemental Figures and Figure Legends**

**
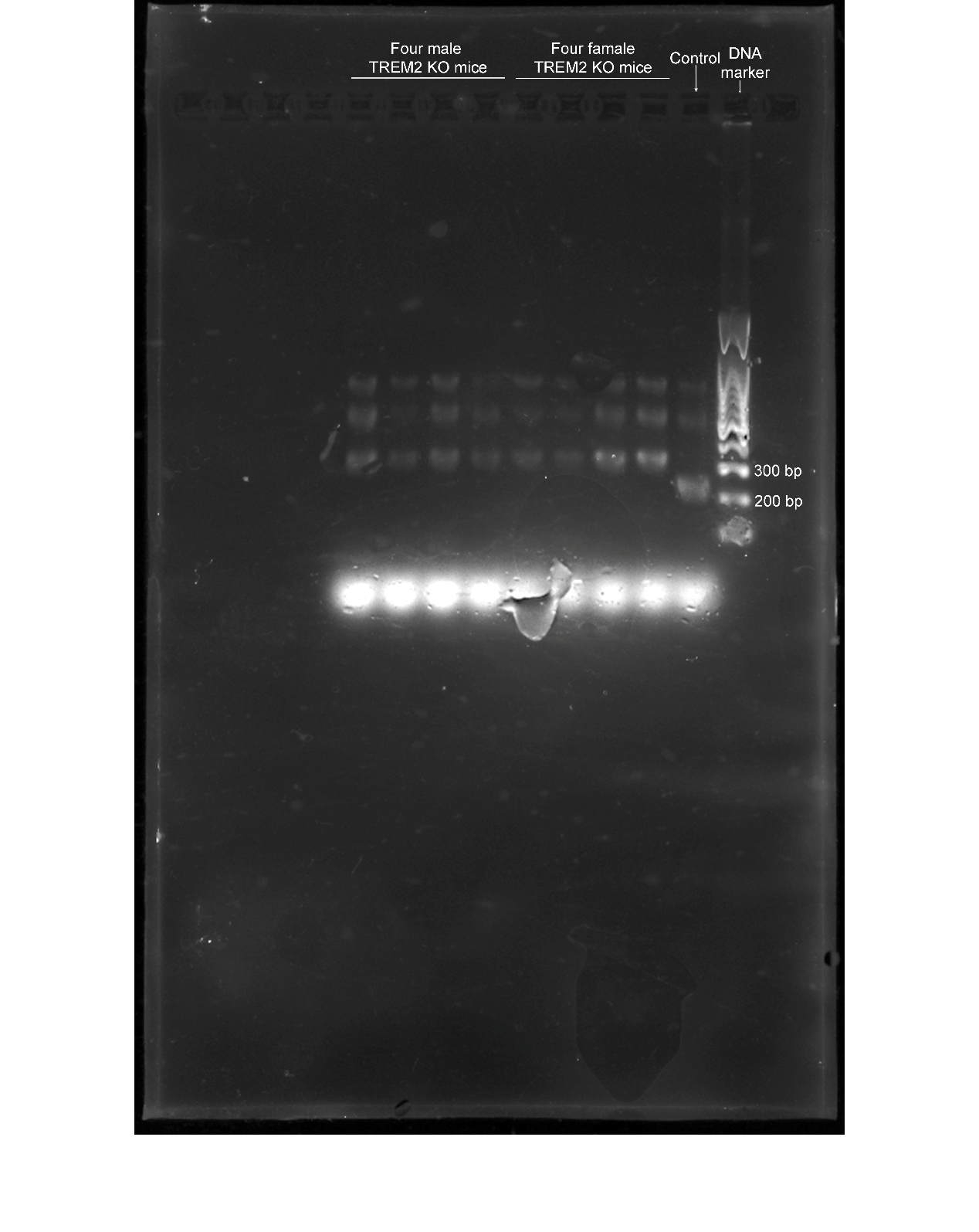
**

**Suppl Fig. 1 Image of TREM2 genotype identification**

The mutant band is localized at approximate 300 bp and the WT band is localized at approximate 200 bp as shown in the image. Consequently, the genotype of F0 mice were all homozygote TREM2 KO. Adult male TREM2 KO mice used in this study were offspring of these F0 generations. Besides, the quantitative transcriptome analysis and the western blot analysis all showed that TREM2 was abolished in TREM2 KO mice (see text Fig. 9D and Fig. 9F).


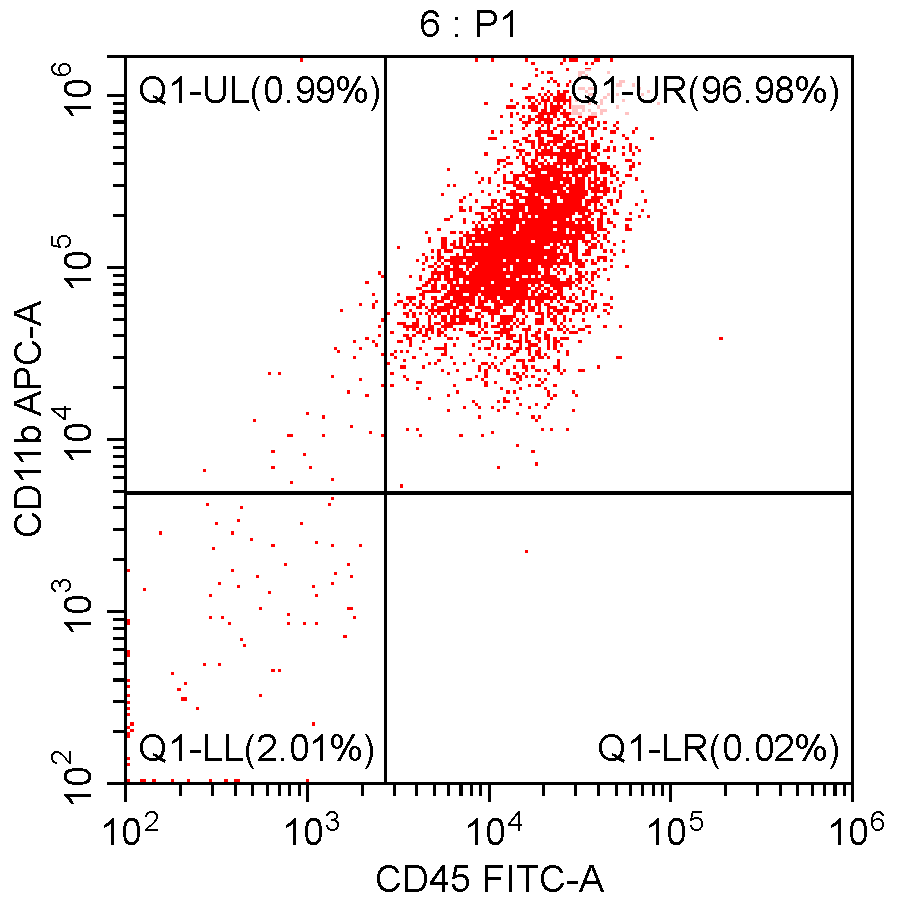


**Suppl Fig. 2 Image of microglial purity by flow cytometry**

FITC-labeled anti-CD11b monoclonal antibody and APC-labeled anti-CD45 monoclonal antibody were used to mark the microglia, then, flow cytometry was performed to identify the purity of the microglia. The results demonstrated that we could extract microglia with a purity of approximately 97%.


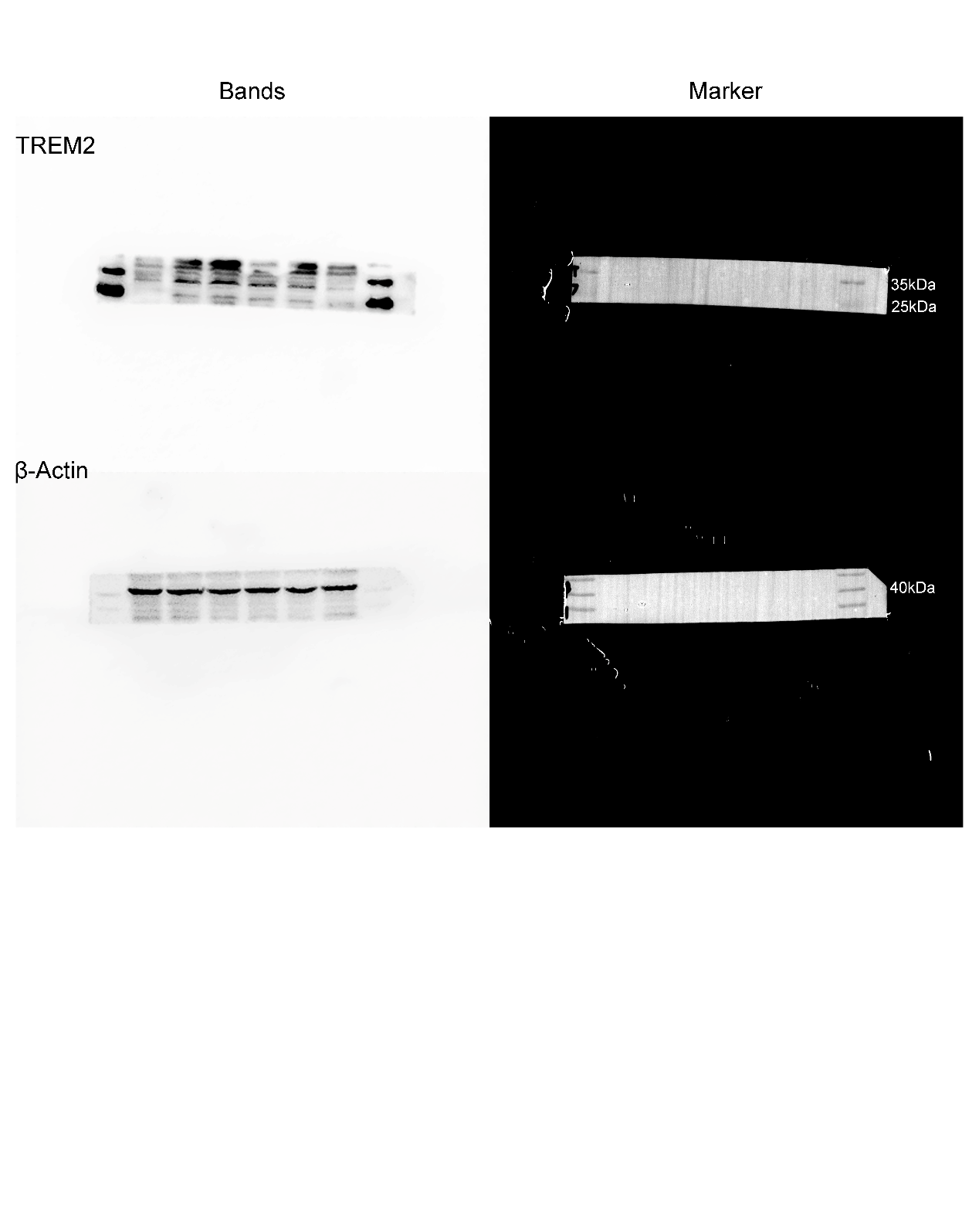


**Suppl Fig. 3 Raw western blot bands in Fig. 1**


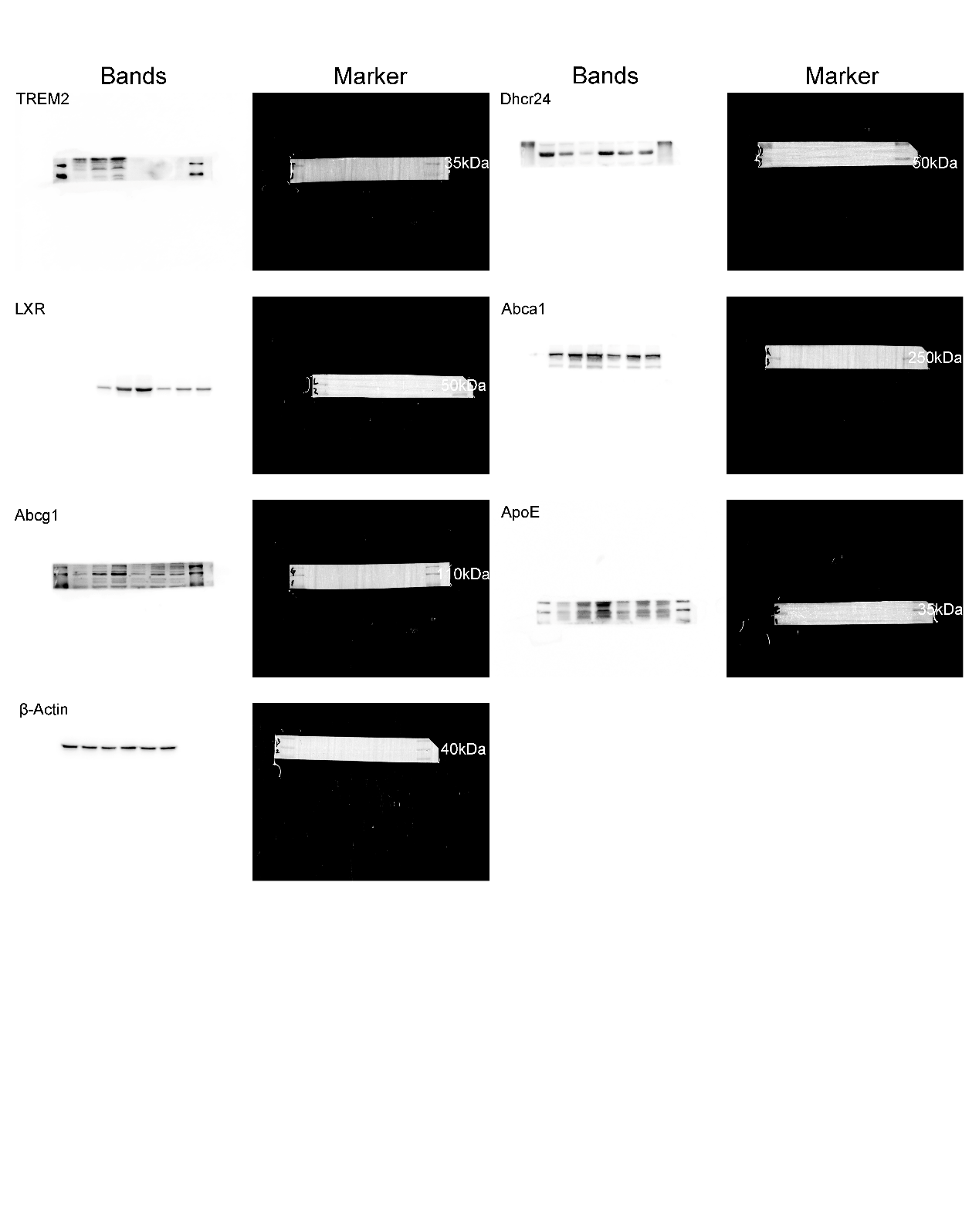


**Suppl Fig. 4 Raw western blot bands in Fig. 9**
